# Palliative radiotherapy after oesophageal cancer stenting (ROCS): a multicentre, open-label, phase 3 randomised controlled trial

**DOI:** 10.1016/S2468-1253(21)00004-2

**Published:** 2021-02-19

**Authors:** Douglas Adamson, Anthony Byrne, Catharine Porter, Jane Blazeby, Gareth Griffiths, Annmarie Nelson, Bernadette Sewell, Mari Jones, Martina Svobodova, Deborah Fitzsimmons, Lisette Nixon, Jim Fitzgibbon, Stephen Thomas, Anthony Millin, Tom Crosby, John Staffurth, Christopher Hurt

**Affiliations:** a Tayside Cancer Centre, Ninewells Hospital, Dundee, UK; b Division of Population Medicine, Cardiff University, Cardiff, UK; c Marie Curie Palliative Care Research Centre, Cardiff University, Cardiff, UK; d Centre for Trials Research, Cardiff University, Cardiff, UK; e Bristol Centre for Surgical Research, National Institute for Health Research Bristol and Weston Biomedical Research Centre, Bristol University, Bristol, UK; f University of Southampton, Southampton, UK; g Swansea Centre for Health Economics, Swansea University, Swansea, UK; h Radiotherapy Trials Quality Assurance Group, Velindre Cancer Centre, Cardiff, UK; i Velindre University NHS Trust, Cardiff, UK

## Abstract

**Background:**

Patients with advanced oesophageal cancer have a median survival of 3–6 months, and most require intervention for dysphagia. Self-expanding metal stent (SEMS) insertion is the most typical form of palliation in these patients, but dysphagia deterioration and re-intervention are common. This study examined the efficacy of adjuvant external beam radiotherapy (EBRT) compared with usual care alone in preventing dysphagia deterioration and reducing service use after SEMS insertion.

**Methods:**

This was a multicentre, open-label, phase 3 randomised controlled trial based at cancer centres and acute care hospitals in England, Scotland, and Wales. Patients (aged ≥16 years) with incurable oesophageal carcinoma receiving stent insertion for primary management of dysphagia were randomly assigned (1:1) to receive usual care alone or EBRT (20 Gy in five fractions or 30 Gy in ten fractions) plus usual care after stent insertion. Usual care was implemented according to need as identified by the local multidisciplinary team (MDT). Randomisation was via the method of minimisation stratified by treating centre, stage at diagnosis (I–III *vs* IV), histology (squamous or non-squamous), and MDT intent to give chemotherapy (yes *vs* no). The primary outcome was difference in proportions of participants with dysphagia deterioration (>11 point decrease on patient-reported European Organisation for Research and Treatment of Cancer quality of life questionnaire-oesophagogastric module [QLQ-OG25], or a dysphagia-related event consistent with such a deterioration) or death by 12 weeks in a modified intention-to-treat (ITT) population, which excluded patients who did not have a stent inserted and those without a baseline QLQ-OG25 assessment. Secondary outcomes included survival, quality of life (QoL), morbidities (including time to first bleeding event or hospital admission for bleeding event and first dysphagia-related stent complications or re-intervention), and cost-effectiveness. Safety analysis was undertaken in the modified ITT population. The study is registered with the International Standard Randomised Controlled Trial registry, ISRCTN12376468, and ClinicalTrials.gov, NCT01915693, and is completed.

**Findings:**

220 patients were randomly assigned between Dec 16, 2013, and Aug 24, 2018, from 23 UK centres. The modified ITT population (n=199) comprised 102 patients in the usual care group and 97 patients in the EBRT group. Radiotherapy did not reduce dysphagia deterioration, which was reported in 36 (49%) of 74 patients receiving usual care versus 34 (45%) of 75 receiving EBRT (adjusted odds ratio 0·82 [95% CI 0·40–1·68], p=0·59) in those with complete data for the primary endpoint. No significant difference was observed in overall survival: median overall survival was 19·7 weeks (95% CI 14·4–27·7) with usual care and 18·9 weeks (14·7–25·6) with EBRT (adjusted hazard ratio 1·06 [95% CI 0·78–1·45], p=0·70; n=199). Median time to first bleeding event or hospital admission for a bleeding event was 49·0 weeks (95% CI 33·3–not reached) with usual care versus 65·9 weeks (52·7–not reached) with EBRT (adjusted subhazard ratio 0·52 [95% CI 0·28–0·97], p=0·038; n=199). No time versus treatment interaction was observed for prespecified QoL outcomes. We found no evidence of differences between trial group in time to first stent complication or re-intervention event. The most common (grade 3–4) adverse event was fatigue, reported in 19 (19%) of 102 patients receiving usual care alone and 22 (23%) of 97 receiving EBRT. On cost-utility analysis, EBRT was more expensive and less efficacious than usual care.

**Interpretation:**

Patients with advanced oesophageal cancer having SEMS insertion for the primary management of their dysphagia did not gain additional benefit from concurrent palliative radiotherapy and it should not be routinely offered. For a minority of patients clinically considered to be at high risk of tumour bleeding, concurrent palliative radiotherapy might reduce bleeding risk and the need for associated interventions.

**Funding:**

National Institute for Health Research Health Technology Assessment Programme.

**Research in context**

**Evidence before this study**

Before study commencement we searched Medline and the Cochrane Database of Systematic Reviews for prospective trials of dysphagia palliation interventions published from Jan 1, 1995, to Jan 1, 2011, using the terms “dysphagia”, “stent”, “oesophageal cancer OR carcinoma”, “radiotherapy” and “brachytherapy”. We included randomised controlled trials (RCTs), both masked and unmasked, reported in English. Studies of interest were those with patients with incurable primary squamous cell carcinoma or adenocarcinoma of the oesophagus or oesophagogastric junction. We identified a UK National Institute for Health Research Health Technology Assessment and a Cochrane systematic review confirming the efficacy of self-expanding metal stents (SEMS) in relieving dysphagia. These studies showed that efficacy of SEMS alone was limited by early problems with pain, decline in general aspects of quality of life (QoL), and later complications such as haemorrhage, tumour overgrowth, and recurrent dysphagia within 12 weeks. They called for evidence of interventions in combination with a stent to improve outcomes, and evidence on QoL and cost-effectiveness. These findings were confirmed by a further systematic review during the present study period. We identified two studies of brachytherapy versus stenting that showed longer dysphagia-free survival and more stable health-related QoL with brachytherapy. The literature search confirmed low access to brachytherapy for this patient group across the UK. A search of ClinicalTrials.gov did not reveal any current prospective studies of the more widely available palliative external beam radiotherapy (EBRT) in combination with stenting for improving dysphagia outcomes.

**Added value of this study**

We believe that the present study is the first sufficiently powered RCT to test the efficacy of palliative EBRT in combination with stenting for improving dysphagia outcomes in advanced oesophageal cancer, and the first RCT to show an effect of EBRT on tumour bleeding risk. It also provides prospective data on the effect of radiotherapy on re-interventions and service use. The addition of radiotherapy after oesophageal stent insertion does not reduce dysphagia deterioration and adds significantly to the cost of treatment. Reduction of upper gastrointestinal bleeding was observed in patients randomly assigned to receive radiotherapy after stent insertion, which warrants further investigation. This trial provides detailed findings on the poor outcomes in this patient group, which are rarely the focus of multicentre prospective research.

**Implications of all the available evidence**

Patients with advanced oesophageal cancer and dysphagia have limited treatment options and outcomes are often poor. Re-interventions are common following stent insertion. This study shows that when stent insertion is required to palliate dysphagia, most patients will not additionally benefit from—and should not be routinely offered—locoregional radiotherapy. Radiotherapy might be reserved for a minority of patients at high risk of tumour bleeding, but this strategy should be balanced against the burdens of treatment. Our study highlights the considerable unmet needs of this patient group. We hope our findings will challenge upper gastrointestinal services to establish better evidence in this field on other combination therapies and how multidisciplinary support can help patients and families negotiate symptom burden and improve QoL.

## Introduction

In the UK, data from 2015–18 showed more than 9000 new cases of oesophageal cancer and approximately 8000 deaths due to oesophageal cancer each year. Worldwide in 2012, more than 450 000 new cases were diagnosed, with greater than 80% of new cases and deaths occurring in low-income and middle-income nations.^1^ Most patients present with incurable disease. For patients with advanced disease, median survival is 3–6 months,^2^ with the majority requiring intervention for dysphagia.^3^, ^4^

Although the ideal intervention for dysphagia palliation has not been defined, options include chemical and thermal ablation, self-expanding metal stents (SEMS), and radiotherapy and chemotherapy alone or in combination. Evidence suggests that in advanced incurable oesophageal cancer, SEMS insertion is an appropriate intervention for rapid dysphagia relief.^5^, ^6^, ^7^ Brachytherapy might represent an appropriate alternative^8^, ^9^ but is rarely accessible in UK National Health Service (NHS) settings,^10^ and is unavailable in most low-income countries where the incidence of advanced oesophageal cancer is highest.^1^ Although SEMS is widely implemented for first-line management of dysphagia in the UK, the efficacy of SEMS alone is limited by early problems with pain, a decline in general aspects of quality of life (QoL), and later complications such as haemorrhage and tumour overgrowth.^5^, ^6^, ^7^ Median time to recurrent dysphagia in stent comparator^11^, ^12^ and brachytherapy studies^8^ is 11–12 weeks and it has profound effects on independence, social function, and QoL. Hospitalisation and re-intervention account for most stent-related costs,^5^ imposing a substantial burden on both NHS resources and a vulnerable population with a median overall survival of 3–6 months. In line with Cochrane review research recommendations,^6^, ^7^ combination of SEMS with other treatments might reduce costs and patient burden by reducing adverse events and re-interventions at a time when patients are approaching the last weeks of life.

The Radiotherapy after Oesophageal Cancer Stenting (ROCS) study was developed in response to a UK National Institute for Health Research (NIHR) call for research proposals into aspects of palliation, and aimed to address uncertainties in the evidence base for interventions combined with SEMS. External beam radiotherapy (EBRT) is rarely used in the UK as a monotherapy for rapid dysphagia relief, but its use in the immediate post-stent period has not been rigorously studied. This study addresses the efficacy of adjuvant EBRT compared with SEMS alone in reducing the risk of dysphagia deterioration, and improving QoL and patterns of service use in patients with advanced oesophageal cancer.

## Methods

### Study design and participants

The ROCS study was designed as a multicentre, parallel-arm, open-label, phase 3 randomised controlled trial with an internal pilot phase examining recruitment. It was done in cancer centres and acute care hospitals across Scotland, England, and Wales.

A description of the original trial protocol has previously been published.^13^ A summary of changes to the original ROCS protocol are provided in the appendix (p 2). The final trial protocol is available online. Ethics approval for the study was given by the Wales Research Ethics Committee 2 in October, 2012 (reference 12/WA/0230).

Participants were patients with incurable oesophageal carcinoma (histologically confirmed excluding small cell carcinoma; or clinical or radiological evidence of invasive tumour and at least high-grade dysplasia of a non-small cell type on histology), already referred for a stent as primary palliation of dysphagia by members of the local upper gastrointestinal multidisciplinary team (MDT), age 16 years or older, having an expected survival of at least 12 weeks, deemed clinically able by the MDT to tolerate radiotherapy, having completed a baseline (post-consent) European Organisation for Research and Treatment of Cancer (EORTC) quality of life questionnaire-oesophagogastric module (QLQ-OG25),^14^ unsuitable for radical treatment (oesophagectomy or radical chemoradiotherapy) because of patient choice or medical reasons, and with the ability to provide written informed consent. We excluded patients planned to receive endoscopic treatment of the tumour, other than dilatation, in the peri-stent period (except for required emergency interventions), those with a tumour length of greater than 12 cm (or tumour growth within 2 cm of the upper oesophageal sphincter), those with presence of a tracheo-oesophageal fistula, or pacemaker in the proposed radiotherapy field, those who had previous radiotherapy to the area of the proposed radiotherapy field, and those who were pregnant. Patients in whom brachytherapy or EBRT was already planned after stent insertion were not included as we were concerned that further addition of trial radiotherapy might increase the risk of toxicity. The number of such patients was small (brachytherapy accounts for <2% of dysphagia interventions in the UK and EBRT is rarely used for immediate dysphagia relief in the UK in the participants of interest^10^). Patients were approached and randomly assigned either before or after stent insertion to allow pragmatic accommodation within the clinical pathway.

### Randomisation and masking

Patients were randomly assigned 1:1 to receive EBRT (plus usual care) or usual care alone after stenting by the method of minimisation with a random element (80:20) via a central telephone randomisation system developed by, and based at, the Centre for Trials Research at Cardiff University (Cardiff, UK). Minimisation was stratified to ensure balanced treatment allocation by a number of potential confounding factors: treating centre, stage at diagnosis (I–III *vs* IV), histology (squamous or non-squamous), and MDT intent to give chemotherapy (yes or no). Participants were enrolled and assigned their trial group by the local principal investigator or research practitioner. The research practitioner was responsible for subsequent follow-up data collection. The study was necessarily open label and neither the patients nor the treating clinicians were blinded to treatment allocation. However, classification of some events was blinded, detailed herein.

### Procedures

SEMS insertion was done in the EBRT group and the usual care group as per standard procedures at each centre. Stent type and length were determined by the treating clinician. When possible, the stent length was chosen to ensure that at least 2 cm of normal oesophagus was covered by the stent above and below the tumour.

Usual care was implemented in both groups according to local MDT practice to include, as needed, post-stent dietetic advice, referral for palliative and supportive care interventions (eg, blood transfusion and supportive oncology), and community-based health-care and social-care follow-up.

In the EBRT group, the study protocol mandated that radiotherapy begin within 4 weeks of stent insertion and preferably 2 weeks. Treatment dose was prespecified at each centre, preferably 20 Gy in five fractions over 1 week or, at the treating clinician's discretion, 30 Gy in ten fractions over 2 weeks. Treatment was administered according to each centre's normal radiotherapy procedures without corrections for inhomogeneity in dose calculation. In the event of severe radiotherapy side-effects or treatment machine unavailability, gaps in treatment of up to 7 calendar days were allowed. If the patient missed more than 7 consecutive calendar days during radiotherapy treatment, then they were withdrawn from the trial and further treatment given at the clinician's discretion. Radiotherapy quality assurance was monitored by the NIHR Radiotherapy Trial Quality Assurance Group.

Follow-up at home was planned 1 week after stent insertion (before any radiotherapy; forming the baseline measurement for the primary outcome), and every 4 weeks thereafter for up to 1 year (finishing when the last patient enrolled had been followed up for 12 weeks). At each timepoint, participants completed the EORTC QLQ-OG25, EORTC QLQ core 30 (QLQ-C30; version 3.0),^15^, ^16^ and EuroQol 5D questionnaire 3 level (EQ-5D-3L).^17^, ^18^ Data were also collected on WHO performance status, stent complications, toxicities (as per the National Cancer Institute Common Terminology Criteria for Adverse Events [CTCAE] version 4.03), other treatments, and health-care and social-care resource use. If a home visit was not possible, or if patients preferred, data were collected by phone. Additionally, phone calls every 4 weeks were introduced midway between home visits to maximise capture of the primary outcome data only. Serious adverse events were collected in real time via a designated contact service from time of informed consent until 60 days after stent insertion.

During the pilot phase, an embedded qualitative study based on longitudinal interviews explored the feasibility of trial recruitment in a subset of patients during their first 8 weeks after stent insertion, relating to patient experience of the trial and recruitment process, the effect of trial interventions on daily life, and the experiences of living with advanced oesophageal cancer. 30 longitudinal interviews in 15 patients (nine in the EBRT group and six in the usual care group) were done in their first 8 weeks of trial involvement. Interviews were analysed with the Braun and Clarke framework for thematic analysis.^19^ Full results of this qualitative study will be reported separately.

### Outcomes

The primary outcome was deterioration in a dysphagia event within 12 weeks after stent insertion, defined as: two consecutive deteriorations of more than 11 points from baseline^20^ in patient-reported dysphagia score on the EORTC QLQ-OG25, with the first being taken as the event timepoint (consecutive deteriorations were specified because patients undergoing radiotherapy might temporarily show worsening of dysphagia secondary to radiation-induced oesophagitis); one deterioration and no more data possible (patient withdrew completely or died before next visit); one deterioration and patient missing on the next visit, with patient withdrawal or death within 4 weeks of the missed visit; additional dysphagia-related primary events consistent with the relevant change in dysphagia score (additional stent insertion, hospital admission for dysphagia, overgrowth or undergrowth of the stent, grade ≥3 dysphagia [CTCAE v4.03], or additional radiotherapy to the oesophagus or stent region; assessed and confirmed by the chief investigators [DA and AB] as tumour related and reviewed by an independent gastroenterologist, all masked to treatment group, as a dysphagia-related event); or death from any cause. In patients showing deterioration at one assessment but with missing data at a subsequent assessment, deterioration was timed at the previous assessment.

Secondary outcomes were overall survival, QoL (including WHO performance status), morbidity (upper gastrointestinal-related bleeding event or hospital admission for a bleeding event, first dysphagia-related stent complication, or re-intervention), dysphagia deterioration-free survival (DDFS), post-stent chemotherapy or additional radiotherapy, patient experience (to be reported in detail elsewhere), and cost effectiveness. Overall survival was calculated from the date of stent insertion to the date of death from any cause. QoL was measured with the EORTC QLQ-C30, EORTC QLQ-OG25, and EQ-5D-3L. The prespecified main patient-reported outcome items were the global health score from the QLQ-C30 and four scales from the EORTC QLQ-OG25: odynophagia, pain or discomfort, eating restrictions, and eating in front of others. Upper gastrointestinal bleeding events were confirmed by the chief investigators who were masked to the study group and reviewed by a masked independent gastroenterologist. These events could include blood transfusion, haematemesis, other descriptions of upper gastrointestinal haemorrhage or bleeds, or interventions related to bleeding (such as argon plasma coagulation or additional radiotherapy). If there was no clinical evidence that anaemia was due to a bleed then it was not considered. Data on treatment with antiplatelet drugs, anticoagulants, and nonsteroidal anti-inflammatory drugs other than aspirin were collected at each clinical assessment visit. Stent complications were defined as re-stenting, repeat endoscopy, overgrowth or undergrowth of the stent, stent blockage, stent fracture, stent slippage, and stent-related pain (grade ≥2 on the CTCAE v4.03). Dysphagia-related stent events (blindly assessed) only influenced the primary outcome if an event had not already been identified in the patient-reported OG25 assessments. Re-interventions (dyphagia-related or not related) were defined as additional stent insertion, stent removal, endoscopic intervention (including laser therapy and alcohol injection), and other palliative radiotherapy (including brachytherapy and additional EBRT for dysphagia). For monitoring of safety, toxicity was measured throughout follow-up with the CTCAE v4.03.

### Statistical analysis

Originally the sample size calculation was based on a time-to-event analysis for the primary endpoint requiring 496 participants. However, during the recruitment phase of the trial, in view of lower than expected eligible patient numbers and substantial missing data after 12 weeks reflecting patient deterioration, the independent data monitoring committee (IDMC) recommended a revised sample size calculation, based on comparison of proportions with an event by week 12 rather than a time-to-event analysis. No early analysis was done that might have influenced this recommendation. To detect a reduction in the proportion of patients with deterioration from 40% to 20% required 164 patients (82 patients per group; 80% power at a two-sided α level of 5%), with a total of 220 to be recruited to allow for 25% loss to follow-up. This difference in proportions was larger than that for the original sample size sought but was in line with the difference sought in other studies of stent or non-stent interventions for malignant dysphagia.^8^, ^21^, ^22^ The changes were approved by the independent trial steering committee and ratified by the funder following further independent review.

All statistical analyses followed a predefined statistical analysis plan agreed with the IDMC. Our modified intention-to-treat (ITT) population was defined as all patients who had a stent inserted (otherwise no benefit from radiotherapy was expected) and returned a baseline EORTC QLQ-OG25 (an eligibility criteria). The per-protocol (PP) population was defined as the subgroup of the modified ITT population that was alive and had not withdrawn from trial treatment at 4 weeks after stent insertion, and, in the EBRT arm, had received at least one fraction of radiotherapy to compare those who could have received radiotherapy in the usual care arm with those who did in the EBRT arm.

Analysis of the primary binary endpoint of deterioration in dysphagia symptoms by 12 weeks was primarily done in the modified ITT population with complete case data. Complete cases were defined as having complete data for the dysphagia subscale of the QLQ-OG25 questionnaire at baseline, week 4, week 8, and week 12, or having died with complete data before week 12. In the absence of a documented dysphagia-related event, missing dysphagia scores between two non-event dysphagia scores were assumed to be no event. Multivariable logistic regression was used to adjust for randomisation stratification factors and obtain odds ratios (ORs) and 95% CIs for any treatment effect in the primary analysis and all sensitivity analyses. We did three sensitivity analyses: using the same complete case population but treating death by 12 weeks without earlier deterioration as no deterioration; imputing missing data using a best-case scenario that assumed no deterioration in a missing QLQ-OG25 form immediately before an QLQ-OG25 form that showed deterioration (or a dysphagia-related primary event), or that assumed no deterioration in a missing QLQ-OG25 form immediately before death; and imputing missing data using a worst-case scenario that assumed deterioration in a missing QLQ-OG25 form immediately before an QLQ-OG25 form that showed deterioration (or a dysphagia-related primary event), or that assumed deterioration in a missing QLQ-OG25 form immediately before death. As further sensitivity analyses, all analyses were repeated in the PP population.

As a secondary endpoint per IDMC guidance, DDFS was calculated in the ITT population from the date of stent insertion to the date of deterioration in dysphagia (as per the primary outcome definition). We analysed overall survival and DDFS using Kaplan-Meier plots and Cox regression (with the usual care group as the reference for the treatment effect measured by hazard ratios [HRs] and 95% CIs), with patients without events being censored at the time of last contact and adjusted for randomisation stratification factors with treating centre included as a shared frailty. We tested the model fit and assumptions using Cox-Snell residuals and Schoenfeld's global test.

QoL data and WHO performance status scores, prespecified in the statistical analysis plan, were analysed by the same method: the distributions of the variables were tested for normality with the Shapiro-Wilk test, kernel density, normal probability, and normal quantile plots, and either mean scores (or median scores if the was evidence of non-normality) plotted accordingly. Box plots were used to show the median, IQR, upper and lower adjacent values, and any outliers (per STATA 16 procedures) as dots, at each timepoint. Mean values were plotted with 95% CIs against time. Linear mixed models were used to compare differences between trial groups for each subscale or single item on the EORTC QLQ-C30, EORTC QLQ-OG25, and WHO performance status. We included time as a categorical covariate using the week of observation from week 1 to week 16, after which the proportion of missing data became too high (>30% of randomly assigned patients returning questionnaires). If an intermediate value was missing, the corresponding time was skipped. Covariates included trial group, age, time 0 score, and randomisation stratification factors. The mixed model residuals were tested for normality.

Time to first morbidity event was compared between trial groups by competing risks regression (used to calculate subhazard ratios and 95% CIs), with death as a competing risk, adjusted for randomisation stratification factors, and with cumulative incidence functions plotted by trial group and median time to event calculated with the stci command in STATA. Treatment-emergent grade 3–4 toxicity was reported in the modified ITT population. Risk ratios were calculated in a post-hoc analysis to compare rates of toxicities and post-stent chemotherapy or additional radiotherapy between treatment arms.

We assessed cost-effectiveness from a UK NHS and Personal Social Services perspective using a combined decision tree and Markov model (appendix p 33), comparing the costs of usual care alone to EBRT over a time horizon of 12 weeks after stent insertion (extended to 12 months in a sensitivity analysis). We considered the intervention implementation costs of EBRT and the cost of subsequent health and social care resource use (primary, secondary, hospice, and social care including any cancer treatment and medications received). Health and social care resource use was captured with a Client Service Receipt Inventory. As the analysis was less than 12 months, discounting was not applied. The model calculated the incremental cost per quality-adjusted life-year (QALY) gained based on dysphagia data, health-care resource use, and health utilities derived from the EQ-5D-3L responses collected at the follow-up points every 4 weeks and the UK EQ-5D value set.^23^ Costs were applied by calculating number of patients in each stage of the model and multiplying by mean cost for that stage. In the base-case analysis, patient-level data was used to populate the model with the first 12 weeks of data. The health-care costs in primary, secondary and social care for both EBRT and control groups post-randomisation were summated and mean absolute cost difference per patient (including 95% CIs and p values) were calculated with SPSS (version 26). Independent sample *t*-tests were used for comparisons with a 5% significance level. For the 12-month time horizon, the model structure remained the same. However, costs, utilities, and transition probabilities between the health states of stable or worsening dysphagia were updated to include the data from 13–52 weeks. Where data were missing, mean patient-level interpolation was used.

Sensitivity analyses were undertaken to test the robustness of the cost utility analysis considering the uncertainty in input parameters such as costs and outcomes and in different scenarios. In a deterministic, univariate sensitivity analysis, we changed intervention and health-care costs and outcomes individually within plausible ranges (using 10%, 20%, and 30% of the mean value). Scenario analyses were used to test different assumptions and recalculate the incremental cost per QALY gained (eg, based on different populations: complete cases and all available cases). The time horizon was also extended to 12 months to explore longer term effects of the intervention. In a probabilistic sensitivity analysis, we used non-parametric bootstrapping to address joint parameter uncertainty and assess the effect on the incremental cost during 1000 simulations, undertaken with random sampling from distributions of costs and outcomes (with replacement).

Detailed statistical methods are presented in the protocol.^13^ In all analyses, a p value of less than 0·05 was considered to indicate significance. All statistical analyses were done with STATA 16. This study is registered as an International Standard Randomised Controlled Trial, ISRCTN12376468, and with ClinicalTrials.gov, NCT01915693.

### Role of the funding source

The funders of the study had no role in study design, data collection, data analysis, data interpretation, or writing of the report.

## Results

**Figure 1:**
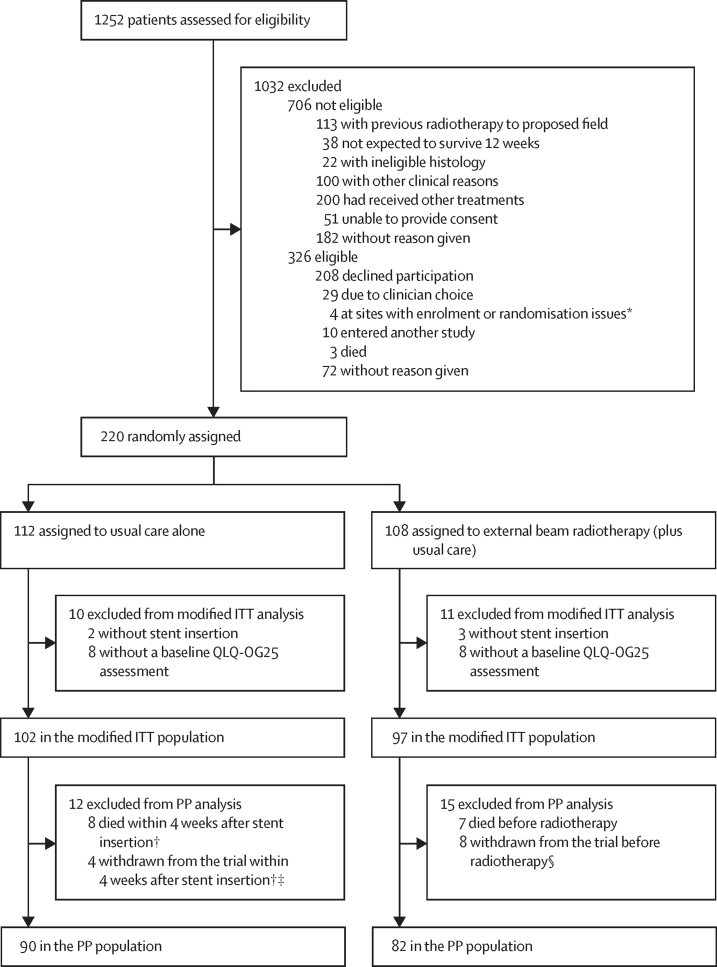
Inclusion of patients receiving a stent for primary palliation of dysphagia ITT=intention-to-treat. QLQ-OG25=quality of life questionnaire-oesophagogastric module. PP=per-protocol. *Research staff unavailable. †The number of weeks before radiotherapy should have been started. ‡Two patients withdrawn due to patient choice; one patient uncontactable; and one patient relocated. §Seven patients withdrawn due to patient choice; and one at clinician request.

1252 patients were screened, 546 were found to be eligible, and 220 were enrolled and randomly assigned at 23 hospital sites (appendix p 21) between Dec 16, 2013, and Aug 24, 2018. Of these patients, 112 were allocated to receive usual care alone and 108 to receive adjuvant EBRT (plus usual care) after stent insertion (figure 1). The modified ITT population comprised 102 patients in the usual care group and 97 in the EBRT group. Unless otherwise stated, all results described herein are for the modified ITT population. Baseline characteristics are shown in table 1 and details of stents used are given in the appendix (p 3). The randomisation stratification factors of tumour histology, stage at diagnosis, and intended chemotherapy after stent insertion (and treating centre [data not shown]) were well balanced between trial groups as were other baseline characteristics.

**Table 1:** Baseline demographic and clinical characteristics in the modified ITT population

	**Usual care group (n=102)**	**External beam radiotherapy group (n=97)**
**Randomisation time point**		
Before stent insertion	39 (38%)	36 (37%)
After stent insertion	63 (62%)	61 (63%)
Age, years	73·5 (65·4–81·5); 102	72·0 (65·3–79·9); 97
**WHO performance status**		
0	10 (10%)	10 (10%)
1	61 (60%)	59 (61%)
2	27 (26%)	27 (28%)
3	4 (4%)	1 (1%)
**Tumour type**		
Adenocarcinoma	68 (67%)	61 (63%)
Squamous	33 (32%)	34 (35%)
Undifferentiated or other	1 (1%)	2 (2%)
Overall length of primary tumour (endoscopic assessment)*		
Measured length, cm	5·0 (4·0–7·0); 64	6·0 (4·0–8·0); 57
Estimated length, cm	6·0 (4·5–8·0); 28	7·0 (5·0–8·0); 33
Measured or estimated length, cm	5·9 (4·0–7·0); 92	6·0 (4·5–8·0); 90
Missing	10 (10%)	7 (7%)
Alternative method for assessing length*		
PET	5 (5%)	7 (7%)
CT	23 (23%)	23 (24%)
Missing	0	3 (3%)
**Site of predominant tumour**		
Upper oesophagus	3 (3%)	3 (3%)
Middle oesophagus	24 (24%)	25 (26%)
Lower oesophagus	75 (74%)	68 (70%)
If lower oesophagus: involvement of GOJ	38 (37%)	38 (39%)
Unknown	0	1 (1%)
**Extension across GOJ (if involvement of GOJ)**		
Siewert type 1	21 (21%)	20 (21%)
Siewert type 2	15 (15%)	13 (13%)
Missing	2 (2%)	5 (5%)
**T stage**†		
0	1 (1%)	0
1	1 (1%)	1 (1%)
2	4 (4%)	7 (7%)
3	61 (60%)	54 (56%)
4	29 (28%)	31 (32%)
Missing	6 (6%)	4 (4%)
**N stage**†		
0	17 (17%)	10 (10%)
1	46 (45%)	46 (47%)
2	20 (20%)	20 (21%)
3	15 (15%)	17 (18%)
Missing	4 (4%)	4 (4%)
**M stage**†		
0	46 (45%)	41 (42%)
1	49 (48%)	50 (52%)
Missing	7 (7%)	6 (6%)
**Overall stage**†		
I–III	51 (50%)	46 (47%)
IV	51 (50%)	51 (53%)
**Previous chemotherapy given**‡		
No	87 (85%)	74 (76%)
Yes	15 (15%)	23 (24%)
**Chemotherapy intended after stent insertion**		
Yes	36 (35%)	34 (35%)
No	66 (65%)	63 (65%)
EQ-5D-3L index scores	0·648 (0·226); 102	0·578 (0·297); 97

Data are n (%), median (IQR); n, or mean (SD); n. ITT=intention to treat. GOJ=gastric-oesophageal junction. EQ5D=EuroQol-5D-3L.

* In addition to endoscopic assessment in some patients.

† At diagnosis.

‡ Details of therapies provided in the appendix p 20.

Compliance with radiotherapy is shown in the appendix (p 4) for patients assigned to EBRT. Of the 97 patients in the EBRT group, 15 (15%) died or were withdrawn from the trial before radiotherapy treatment was given (figure 1). One (1%) participant chose a reduction in the planned radiotherapy from 30 Gy in ten fractions to 15 Gy in five fractions. All remaining participants received the protocol radiotherapy except one (1%) participant who received 8 Gy in one dose as this was the local practice (classified by the trial management group [AB, DA, JB, GG, AN, LN, MS, JF, ST, AM, JS, TC, and DF] as a minor deviation as an appropriate palliative dose; and patient retained in the PP population).

Of the modified ITT population, 74 patients in the usual care group versus 75 patients in the EBRT group had complete data at week 12 for the primary endpoint (table 2). The baseline characteristics of patients with and without complete data were well balanced (appendix p 6). Our primary analysis of complete case data showed no significant difference in the proportion of patients with a dysphagia deterioration event up to 12 weeks after stent insertion between the groups (36 [49%] of 74 patients *vs* 34 [45%] of 75 patients; adjusted OR 0·82 [95% CI 0·40–1·68], p=0·59). The sensitivity analyses treating death as no event, imputing missing data under best-case and worse-case scenarios (table 1), and in the PP population (appendix p 8) consistently found no significant difference.

**Table 2:** Patient status and primary endpoint analyses at 12 weeks after stent insertion in the modified ITT population

	**Usual care group (n=102)**	**External beam radiotherapy group (n=97)**	Adjusted odds ratio*(95% CI)	**p-value**
**Incomplete case data at week 12**				
Withdrawn from the trial before week 12 with no event	6 (6%)	5 (5%)	NA	..
Died before week 12 with incomplete data and no event†	8 (8%)	6 (6%)	NA	..
Alive at week 12 with incomplete data and no event†	14 (14%)	11 (11%)	NA	..
**Reasons for trial withdrawal**				
Participant choice	3 (3%)	4 (4%)	NA	..
On family's behalf via clinical nurse specialist	1 (1%)	0	NA	..
Loss to follow-up	1 (1%)	1 (1%)	NA	..
Relocated	1 (1%)	0	NA	..
**Complete case data at week 12**				
Total with complete data	74 (73%)	75 (77%)	NA	..
Died with complete data	20 (20%)	22 (23%)	NA	..
Alive at week 12 with complete data	54 (53%)	53 (55%)	NA	..
**Primary analysis: complete case data (death without earlier deterioration as an event)**				
Number of primary events or deaths	36/74 (49%)	34/75 (45%)	0·82 (0·40–1·68)	0·59
**Sensitivity analysis: complete case data (death without earlier deterioration as non-event)**				
Number of primary events	21/74 (28%)	21/75 (28%)	1·05 (0·49–2·25)	0·89
**Sensitivity analysis: best-case scenario**				
Total with complete data	90 (88%)	88 (91%)	NA	..
Number of primary events or deaths	40/90 (44%)	36/88 (41%)	0·85 (0·44–1·62)	0·61
**Sensitivity analysis: worst-case scenario**				
Total with complete data	90 (88%)	88 (91%)	NA	..
Number of primary events or deaths	53/90 (59%)	46 (52%)	0·73 (0·38–1·40)	0·35

Data are n (%) unless otherwise specified. NA=not applicable.

* Adjusted for randomisation stratification factors.

† Missing at least one assessment at week 4, week 8, or week 12 and unable to impute a non-deterioration between two adjacent non-deteriorations.

**Figure 2:**
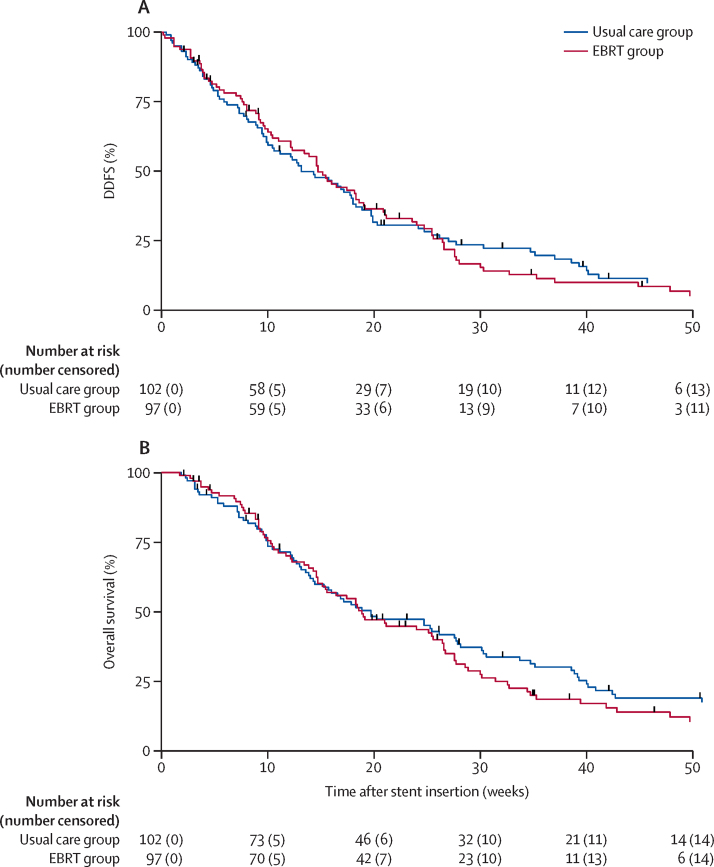
Dysphagia deterioration-free survival (A) and overall survival (B) by trial group up to 1 year DDFS=dysphagia deterioration-free survival. EBRT=external beam radiotherapy.

Analysis of DDFS showed median time to a dysphagia event or death was 13·1 weeks (95% CI 10·0–17·9) in the usual care group and 14·7 weeks (12·1–17·4) in the EBRT group (adjusted HR 0·92 [95% CI 0·68–1·26], p=0·62; figure 2A). We also observed no significant difference in overall survival. Median overall survival was 19·7 weeks (95% CI 14·4–27·7) in the usual care group versus 18·9 weeks (14·7–25·6) in the EBRT group (adjusted HR 1·06 [95% CI 0·78–1·45], p=0·70; figure 2B). Median follow-up in patients alive at the last date of recorded follow-up (Dec 20, 2018) was 22·9 weeks (95% CI 4·0–41·9; n=16) in those receiving usual care versus 22·1 weeks (8·0–34·7; n=15) in those receiving EBRT. The last date of follow-up was March 6, 2019. Total deaths from all causes are summarised in the appendix (p 9).

For EORTC QLQ-C30 and EORTC QLQ-OG25 responses, the proportion of missing data at each timepoint (including withdrawals, deaths, and loss to follow-up) was balanced across trial groups and increased from 0–1% at the first post-stent assessment to 23–25% at week 4, 36–41% at week 8, 49–59% at week 12, and 59–65% at week 16 (appendix pp 10–11). The appendix (pp 12–17) shows the results of the linear mixed models for the EORTC QLQ-C30 and QLQ-OG25 questionnaire scales and WHO performance status. The prespecified main subscales or items of interest were global health (QLQ-C30; appendix p 22), odynophagia (appendix p 22), pain or discomfort (appendix p 23), eating restrictions (appendix p 23), and eating in front of others (appendix p 24; all QLQ-OG25). We found no evidence of a time versus treatment interaction in any of these items (p values >0·05; appendix pp 12–13).

Evidence of a time versus treatment interaction was observed for dysphagia (p=0·013; appendix p 12). The median dysphagia scores were higher (ie, worse swallowing problems and more severe dysphagia) at week 4 in the EBRT group than in the usual care group, but the same by week 8 (appendix p 24). This short-term deterioration was expected, hence the requirement for two successive deteriorations in the definition of the primary endpoint. We also found evidence of a time versus treatment interaction for pain measured by the EORTC QLQ-C30 (p=0·005; appendix p 14). Median pain score was higher at weeks 8, 12, and 16 in the EBRT group (appendix p 25). Furthermore, we observed a time versus treatment interaction for constipation (p=0·009; appendix p 14). The mean score was higher at weeks 8, 12, and 16 in the EBRT group, mirroring the pain scales and possibly related to greater use of analgesia. We also observed significant time–treatment interactions for trouble swallowing saliva, anxiety, body image, and cognitive functioning. We found no evidence of time–treatment treatment interactions for any other QoL scales or WHO performance status (appendix pp 12–17, 25–26).

Median time to first upper gastrointestinal-related bleeding or hospital admission for a bleeding event was longer with EBRT than with usual care (65·9 weeks [52·7–not reached] *vs* 49·0 weeks [95% CI 33·3–not reached]; adjusted subhazard ratio 0·52 [95% CI 0·28–0·97], p=0·038; appendix p 26). Table 3 shows the analysis of upper gastrointestinal-related bleeding events by trial group, for the total study period and up to week 16. Blood transfusion was the most common event, followed by haematemesis and upper gastrointestinal haemorrhage or bleed.

**Table 3:** Upper gastrointestinal-related bleeding events

	**Usual care group (n=102)**	**External beam radiotherapy group (n=97)**
**Overall study period**		
Number of patients with ≥1 event	29 (28%)	16 (16%)
**Number of patients with each type of event**		
Blood transfusion	26 (25%)	13 (13%)
Haematemesis	5 (5%)	6 (6%)
Upper gastrointestinal haemorrhage or bleed	8 (8%)	2 (2%)
Melaena	4 (4%)	0
Argon plasma coagulation due to bleed	0	1 (1%)
Additional radiotherapy due to bleed	1 (1%)	0
Anaemia due to bleed	4 (4%)	2 (2%)
Number of patients with ≥1 event who received antiplatelet drugs*	0	0
Number of patients with ≥1 event who received anticoagulants†	7 (7%)	7 (7%)
Number of patients with ≥1 event who received NSAIDs‡ other than aspirin	4 (4%)	0
**Up to week 16 after stent insertion**		
Number of patients with ≥1 event	19 (19%)	10 (10%)
**Number of patients with each type of event**		
Blood transfusion	13 (13%)	9 (9%)
Haematemesis	4 (4%)	3 (3%)
Upper gastrointestinal haemorrhage or bleed	6 (6%)	1 (1%)
Melaena	3 (3%)	0
Argon plasma coagulation due to bleed	0	0
Additional radiotherapy due to bleed	0	0
Anaemia due to bleed	2 (2%)	2 (2%)
Number of patients with ≥1 event who received antiplatelet drugs*	0	0
Number of patients with ≥1 event who received anticoagulants†	5 (5%)	4 (4%)
Number of patients with ≥1 event who received NSAIDs‡ other than aspirin	2 (2%)	0

Data are n (%). NSAID=non-steroidal anti-inflammatory drug.

* Aspirin or clopidogrel.

† Dalteparin, enoxaparin, rivaroxaban, or other unspecified anticoagulant.

‡ NSAID type not reported separately.

We found no evidence of differences between trial groups in time to first dysphagia-related stent complication or re-intervention event (appendix p 27), including additional stent insertion (appendix p 28), repeat endoscopy (appendix p 29), overgrowth or undergrowth of the stent (appendix p 30), or stent-related pain (appendix p 31).

Table 4 shows the proportions of patients with treatment-emergent grade 3–4 toxicities by trial group between week 0 and week 16. The most common grade 3–4 adverse event was fatigue, reported in 19 (19%) of 102 patients receiving usual care alone and 22 (23%) of 97 receiving EBRT. RRs were calculated (not shown) for each toxicity in a post-hoc analysis, and all of the 95% CIs included 1, but we noted that the point estimates of the RRs were greater than 2 (indicating increased adverse events in the EBRT group) for vomiting and abdominal pain. However, the absolute numbers of patients with abdominal pain were low in both groups (table 3). Three deaths (two in the usual care group, one due to a fall [eventually categorised as hospital-acquired pneumonia after admission for the fall] and one due to myocardial infarction; and one in the EBRT group due to multifocal ischaemic stroke) were reported via the real-time serious adverse event system (appendix p 9), none of which were considered to be related to treatment.

**Table 4:** Participants with grade 3–4 toxicity at weeks 0–16 after stent insertion

	**Usual caregroup (n=102)**	**External beam radiotherapy group (n=97)**
Fatigue	19 (19%)	22 (23%)
Dysphagia	11 (11%)	9 (9%)
Anaemia	10 (10%)	6 (6%)
Anorexia	7 (7%)	9 (9%)
Stent-related pain	6 (6%)	8 (8%)
Nausea	5 (5%)	7 (7%)
Vomiting	4 (4%)	11 (11%)
Upper gastrointestinal haemorrhage	4 (4%)	2 (2%)
Thromboembolic event	3 (3%)	4 (4%)
Bronchopulmonary infection	3 (3%)	3 (3%)
Abdominal pain	2 (2%)	5 (5%)
Oesophageal reflux	2 (2%)	1 (1%)
Dyspepsia	2 (2%)	1 (1%)
Oesophagitis	2 (2%)	0
Abdominal distension	1 (1%)	1 (1%)
Mucositis	1 (1%)	1 (1%)
Fever	1 (1%)	0
Gastritis	1 (1%)	0
Aspiration	0	2 (2%)
Oesophageal fistula	0	1 (1%)*

Data are n (%).

* Detected after stent insertion and participant retained in all analyses.

Additional palliative radiotherapy was received by 20 (20%) of 102 patients in the usual care group and nine (9%) of 97 in the EBRT group (RR 0·47 [95% CI 0·23–0·99], p=0·039; appendix p 18). In the usual care group, 16 (80%) of 20 patients received radiotherapy to the oesophagus, compared with two (22%) of the nine patients in the EBRT group (RR 0·28 [0·08–0·96], p=0·0030). Median dose and fractions were 20 Gy in five fractions in the usual care arm and 8 Gy in one fraction in the EBRT group.

The MDT intended to give chemotherapy after stent insertion to 36 (35%) of 102 patients in the usual care group and 34 (35%) of 97 patients in the EBRT group (table 1). Among these patients, 29 (81%) in the usual care group were given chemotherapy, whereas, less than half (15 [44%]) in the EBRT group were given chemotherapy (RR 0·55 [95% CI 0·36–0·83], p=0·0016; appendix p 19).

In our qualitative study, participants in the EBRT group described whether potential benefits of radiotherapy were worthwhile against additional burdens of hospital attendance and of pain and fatigue. Participants from both study groups revealed ongoing challenges with eating despite stent placement. They suggested that the technical intervention of stenting does not address physical and social eating concerns and symptoms. Information around diet, symptom control, and general medical management throughout the course of the disease was often scarce. Full results of this quantitative analysis will be reported elsewhere.

The costs of health-care and social-care service use in the 12 weeks after stent insertion for the usual care and EBRT groups are reported in table 5 and the appendix (p 32). No significant differences were found in care settings, medication costs, or total care costs (excluding EBRT cost) per patient between groups. Mean EBRT intervention cost was £1297·34 (SD 296·38) per patient, which explained the significant difference in total mean cost (including EBRT cost), of £1831·77 (95% CI 387·43–3276·11, p=0·013) favouring usual care. The base-case cost-utility analysis (12-week time horizon) showed that EBRT was more costly and had lower efficacy than stent only, and thus was not a cost-effective treatment at a willingness-to-pay threshold of £20 000 per QALY gained (appendix p 19). This conclusion did not change when the time horizon was extended to 12 months and when parameters for cost and outcomes were varied in sensitivity analyses (appendix p 20).

**Table 5:** Cost of care resources used per patient in the 12 weeks after randomisation in the modified ITT population

		**Usual care group (n=102)**	**External beam radiotherapy group (n=97)**	**Absolute difference (95% CI)**	**p value**
EBRT cost		NA	£1297·34 (296·38)	..	..
Primary care		£312·01 (410·02)	£310·65 (400·09)	−1·36 (−114·69 to 111·97)	0·98
Secondary care		£3756·21 (4410·26)	£4018·96 (5000·51)	262·75 (−1053·76 to 1579·26)	0·69
Hospice care		£193·67 (746·57)	£150·86 (605·37)	−42·80 (−233·39 to 147·78)	0·66
Social care*		£195·61 (621·18)	£535·17 (1552·36)	339·57 (11·95 to 667·19)	0·042†
Medication		£210·24 (183·42)	£186·52 (186·82)	−23·72 (−75·49 to 28·04)	0·37
Total cost					
	Total cost including EBRT	£4667·73 (4719·99)	£6499·50 (5,593·65)	1831·77 (387·43 to 3276·11)	0·013
	Total cost excluding EBRT	£4667·73 (4719·99)	£5202·16 (5613·63)	534·43 (−912·86 to 1981·73)	0·47

Costs per patient are summarised as mean (SD).

* Visits at home by community nurses or care assistants (eg, to help with personal care).

† This result was no longer statistically significant after Bonferroni-Holm correction for multiple comparisons of total care costs.

## Discussion

Our results show that palliative radiotherapy does not reduce the proportion of patients with recurrent dysphagia at 12 weeks, nor does it improve overall survival or reduce service use. The addition of radiotherapy to stent insertion in advanced oesophageal cancer is therefore not routinely recommended. Patients in the radiotherapy group did have significantly fewer bleeding events, an effect which persisted and increased with time. From a clinical perspective, these findings suggest that radiotherapy could be considered for patients deemed at increased risk of bleeding rather than for all patients, to minimise treatment burden.

ROCS was designed to address gaps in the evidence on improving dysphagia outcomes and NHS and personal burdens in patients with advanced oesophageal cancer,^5^, ^6^, ^7^ with use of a widely available intervention (EBRT) combined with stenting. The choice to assess palliative stent therapy was intended to reflect real-world UK practice, where SEMS is the predominant option for rapid dysphagia relief in advanced oesophageal cancer, accounting for more than 90% of endoscopic and radiological interventions.^10^ ROCS' target population therefore excluded patients with non-severe dysphagia being considered for interventions other than a stent, and those too unwell to have a stent. Although intraluminal brachytherapy might be considered an appropriate alternative to stenting particularly in patients with longer term survival prospects,^7^, ^8^, ^9^ few services in the UK have access to brachytherapy, and it accounts for less than 2% of dysphagia interventions in the NHS.^10^ Similarly, incorporation of endoluminal radiotherapy with a stent as a single modality has been shown to lower dysphagia scores with time compared with a stent alone,^24^ but again, the equipment and expertise is not widely available and cost is likely to be substantially higher.^25^ By contrast, palliative EBRT is widely available across the UK, and at lower cost. The prespecified dose in this study, 20 Gy in five fractions, reflected the most widely used palliative dose used across the UK at the time of study design,^26^ with the 30 Gy in ten fractions dose available if prespecified by the treating clinician. The timing of the primary endpoint at 12 weeks reflects the mean stent patency reported by Homs and colleagues^8^ and other studies,^11^, ^12^ and the median overall survival of around 19 weeks in both groups confirms that our participant population accurately reflects the wider clinical population.^2^ The finding that almost twice as many patients in the control group received their preplanned chemotherapy is noteworthy. This might reflect treatment burden in the radiotherapy group discouraging planned chemotherapy uptake, or could reflect participant or clinician assessment that an active treatment had already been given in the form of radiotherapy.

Exploration of patient experiences in our qualitative study addressed previous gaps in understanding patient and family experiences of advanced oesophageal cancer^5^ and QoL trade-offs.^27^ The results reflect the challenges of their lived experiences of eating restrictions, concerns over nutrition and diet, and a trial and error approach to combating these. These resonate with other findings^28^ and emphasise the need for more structured, proactive multidisciplinary approaches in patients receiving palliative stent therapy.^27^

Although to our knowledge, this study is the first of its kind and has been completed in a patient group with a poor prognosis, it does have some limitations. Originally the sample size calculation was based on a time-to-event analysis for the primary endpoint requiring 496 participants. Due to recruitment and data capture challenges associated with the poor prognosis of the study population, this approach was revised during the study, on advice of the IDMC, to a sample size calculation based on comparison of patient proportions with an event by week 12. Although the revised primary outcome might have affected the ability of the study to detect a true effect for EBRT, the consistency of the results across sensitivity analyses is robust, including the secondary analysis of DDFS. Whether death was treated as an event or not did not alter the primary outcome. In pragmatically allowing two radiotherapy schedules in the EBRT group, we did not aim to seek a difference between doses and the small number of patients receiving 30 Gy precludes any such analysis (64 [78%] of 97 received 20 Gy in five fractions). We also acknowledge the large number of QoL secondary endpoints assessed in this study and associated issues that arise with multiple comparisons, hence we urge caution in the overinterpretation of significant findings found amongst them. Finally, this study was inevitably open-label and some outcome measures could be prone to assessment bias, particularly the adverse events known to be side-effects of radiation. We attempted to mitigate this bias with the use of a comprehensive panel of secondary outcome measures and use of masked assessors to review bleeding events. The baseline QoL assessments (week 1 post-stent) were done after randomisation (and therefore might have been susceptible to bias), but we noted that most scores were balanced between groups at that timepoint.

In summary, the ROCS study confirms that patients with advanced oesophageal cancer requiring a stent to improve dysphagia will not benefit further from the addition of concurrent palliative radiotherapy. In addition, the study provides detailed data on the poor outcomes in these patients, which are rarely the focus of multicentre prospective research. For patients with a long-term prognosis and considered to have a markedly increased risk of tumour bleeding, concurrent palliative radiotherapy might reduce bleeding risk and the need for associated interventions. Future research should focus on alternative, readily accessible interventions that might be effectively combined with stenting or compared as a monotherapy, and effective, multidisciplinary, supportive interventions that address the multidimensional concerns around eating and nutritional intake.

**This online publication has been corrected. The corrected version first appeared at thelancet.com/gastohep on March 11, 2021**

## Data sharing

The Centre for Trials Research at Cardiff University is a signatory of the AllTrials initiative and aims to make research data available when possible, subject to regulatory approvals, any terms and conditions from external providers, patient confidentiality, and all laws concerning the protection of personal information. De-identified participant data and study documents (including the study protocol, statistical analysis plan, participant information sheet, and informed consent form) are generally freely available with publication until Nov 30, 2028, but recipients are expected to acknowledge the original creators in any public use of the data or in publishing research results that are based wholly or in part on the data; anyone requesting access to data will be asked to agree to the terms of the CC BY 4.0 license. We might ask the requestor to cover reasonable cost for preparing and providing the data (for example physical storage and postage, when dataset size makes providing data by electronic means impractical). Please send requests for access to data related to the current study to the open data team (opendata@cardiff.ac.uk) for assessment, providing sufficient detail to uniquely identify the dataset sought and appropriate contact details for the requestor.
