# Correction to *Lancet Gastroenterol Hepatol* 2021; 6: 292–303

**DOI:** 10.1016/S2468-1253(21)00069-8

**Published:** 2021-03-11

*Adamson D, Byrne A, Porter C, et al. Palliative radiotherapy after oesophageal cancer stenting (ROCS): a multicentre, open-label, phase 3 randomised controlled trial.* Lancet Gastroenterol Hepatol *2021; **6:** 292–303*—In this Article, median time to first bleeding event was longer in the EBRT group than in the usual care group and has been corrected throughout the Article: in the Summary Findings, “Median time to first bleeding event or hospital admission for a bleeding event was 49·0 weeks (95% CI 33·3–not reached) with usual care versus 65·9 weeks (52·7–not reached) with EBRT (adjusted subhazard ratio 0·52 [95% CI 0·28–0·97], p=0·038; n=199)”; and in the Results, “Median time to first upper gastrointestinal-related bleeding or hospital admission for a bleeding event was longer with EBRT than with usual care (65·9 weeks [52·7–not reached] *vs* 49·0 weeks [95% CI 33·3–not reached]; adjusted subhazard ratio 0·52 [95% CI 0·28–0·97], p=0·038; appendix p 26)”. Description of fractionation schedules in the Methods has also been amended to “Treatment dose was prespecified at each centre, preferably 20 Gy in five fractions over 1 week or, at the treating clinician's discretion, 30 Gy in ten fractions over 2 weeks”. These corrections have been made to the online version as of March 11, 2021, and the printed version is correct.

