# Supplementary material for: Palliative radiotherapy after oesophageal cancer stenting (ROCS): a multicentre, open-label, phase 3 randomised controlled trial
Source: Lancet Gastroenterol Hepatol. 2021 Feb 19;6(4):292–303. doi: 10.1016/S2468-1253(21)00004-2 (PMC7955283; doi:10.1016/S2468-1253(21)00004-2)
Supplement: Supplementary appendix [file mmc1.pdf]

THE LANCET
Gastroenterology & Hepatology

Supplementary appendix

This appendix formed part of the original submission and has been peer reviewed. We post it as supplied by the authors.

Supplement to: Adamson D, Byrne A, Porter C, et al. Palliative radiotherapy after oesophageal cancer stenting (ROCS): a multicentre, open-label, phase 3 randomised controlled trial. *Lancet Gastroenterol Hepatol* 2021; published online Feb 18. https://doi.org/10.1016/S2468-1253(21)00004-2.

Appendix

Supplementary data for Palliative radiotherapy after oesophageal cancer stenting (ROCS): a multicentre, open-label, phase 3 randomised controlled trial

Supplementary tables

Table S1: Summary of changes to original ROCS protocol approved by Research Ethics Committee

Change to protocol	Date
Exclusion criterion removed: Planned endoscopic treatment of the tumour (e.g. laser) in the immediate peri-stenting period	April 2013
Inclusion criterion added: Patient has completed baseline Quality of Life Questionnaires added in the inclusion criteria	April 2013
Companion patient information sheet and consent form introduced in the qualitative study	September 2014
Dedicated face to face follow up specified as preferred to ensure optimum support for patients; telephone or postal follow up for questionnaire completion also permitted depending on patient's choice.	September 2014
Additional level of withdrawal added – option for participants to stop home visits and questionnaires but follow up	September 2014
Qualitative study added Interviews with research nurses responsible for recruiting patients to the trial.	September 2014
Randomisation allowed within two weeks after stent insertion, but preferably within one week of stent insertion	April 2015
Baseline assessments for those patients consented following stent insertion, ideally baseline assessments will occur within one week, but not more than two weeks, following the procedure.	April 2015
Clarification of time zero due to consent also possible after stent.	April 2015
Secondary outcome added: Determine the haemostatic effect of radiotherapy on tumour bleeding	March 2016
Inclusion criterion of oesophageal carcinoma widened to include clinical and/or radiological evidence of invasive tumour (as agreed by MDT consensus) and at least high grade dysplasia of a non-small cell type on histology	March 2016
Interim phone calls introduced two weeks after stent insertion and four-weekly thereafter to be scheduled half-way between the 4 weekly assessment visits.	March 2016
Dysphagia card was introduced with a list of questions asked during the phone calls.	March 2016
Follow up until death reduced to 1 year, follow up post 1 year is Death CRF only	March 2016
Primary outcome amended:<br>Assess the impact of radiotherapy in addition to stent placement on time to progression of patient-reported dysphagia or other dysphagia-related event in a patient population unable to undergo surgery.	February 2017
Final accrual reduced from original 496 to 220.	December 2017
Primary outcome amended:<br>To assess the impact of radiotherapy in addition to stent placement on difference in event rate of patient-reported dysphagia or other dysphagia-related event at 12 weeks following stent insertion in a patient population unable to undergo surgery.<br>Originally it was intended to perform a time to event analysis. However, in collaboration with the IDMC in response to recruitment difficulties, the primary outcome analysis has been amended and will now be based on proportion of events at week 12. An event is defined as a progression in self-reported dysphagia (see above) or other dysphagia-related event.	December 2017
Follow up for 1 year or up to 3 months after the last patient is recruited whichever comes first	December 2017
New secondary outcome - Measure hospital admission rates	December 2017

Table S2: Stent insertion by trial arm

	UC*	EBRT*
	N=102	N=97
**Type of stent**		
Fully covered stent	31 (30·4)	24 (24·7)
Covered stent with anti-reflux valve	5 (4·9)	3 (3·1)
Partially covered stent	55 (53·9)	58 (59·8)
Partially covered stent with anti-reflux valve	0 (0·0)	1 (1·0)
Uncovered	9 (8·8)	9 (9·3)
Missing	2 (2·0)	2 (2·1)
**Length of stent - median (IQR), n**		
	10·2 (8·0-13·0), 100	10·3 (8·0-13·0), 92
**Dilation required**		
Before stent insertion	4 (3·9)	10 (10·3)
After stent insertion	0 (0·0)	2 (2·1)
Not required	95 (93·1)	80 (82·5)
Missing	3 (2·9)	5 (5·2)
**Radiological imaging used**		
Yes	69 (67·6)	46 (47·4)
No	33 (32·4)	48 (49·5)
Missing	0 (0·0)	3 (3·1)
**Post insertion oesophagogram performed**		
Yes	10 (9·8)	13 (13·4)
No	92 (90·2)	84 (86·6)
If yes, any stent slippage - N(%)		
Yes	0 (0·0)	3 (23·1)
No	10 (100·0)	10 (76·9)
**Number of nights in hospital post stent-median (IQR), n**		
	1·0 (0·0-2·0), 102	1·0 (0·0-2·0), 95
**Acute airway compression**		
Yes	0 (0·0)	0 (0·0)
No	99 (97·1)	96 (99·0)
Missing	3 (2·9)	1 (1·0)
**Oesophageal or other GI tract perforation**		
Yes	0 (0·0)	0 (0·0)
No	101 (99·0)	97 (100·0)
Missing	1 (1·0)	0 (0·0)
**CT scan performed post stent insertion**		
Yes	3 (2·9)	7 (7·2)
No	99 (97·1)	90 (92·8)
**Chest X-ray performed post stent insertion**		
Yes	23 (22·5)	21 (21·6)
No	79 (77·5)	76 (78·4)

*n (%) unless otherwise indicated

Table S3: Compliance with radiotherapy in stent plus radiotherapy arm

	EBRT (N=97)*
**Number of patients receiving radiotherapy**	82 (84·5)
**Number of days from stent insertion to start of radiotherapy-median (IQR; range), n**	
	20 (16-25; 6-61)
**Reasons if radiotherapy not received**	
Withdrew before radiotherapy	7 (7·2)
Died before radiotherapy	8 (8·2)
**Planned dose**	
Planned dose 20Gy in 5 fractions	64 (78·0)
Planned dose 30Gy in 10 fractions	17 (20·7)
Planned dose 8Gy in 1 fractions	1 (1·2)
Reduction to planned dose	1 (1·2)
**Radiotherapy delays**	
Number of delays	10 (12·2)
Reasons for delay	
Toxicity	1 (10·0)
Patient choice	1 (10·0)
Logistical/machine breakdown	3 (30·0)
Been hospitalised hydration	1 (10·0)
Felt ill	1 (10·0)
Weekend break	1 (10·0)
Bank holidays	1 (10·0)
Not known	1 (10·0)
**Number of days delayed-median (IQR; range), n**	
	2·0 (1·0-2·0;1·0-5·0), 10
**Field size (cm)-median (IQR), n**	
X	12·0 (10·0-14·0), 81
Y	11·4 (8·3-14·0), 81
**Field Definition**	
CT Simulator	77 (93·9)
Conventional simulator	5 (6·1)
**Field Arrangement**	
Parallel pair (AP-PA)	76 (92·7)
3D Conformal Radiotherapy	2 (40·0)
4 - field	1 (20·0)
5 - field	1 (20·0)
Conformal "4" field box	1 (20·0)
Missing	1 (1·2)
**Number of beams - median (IQR)**	
	2·0 (2·0-2·0), 82
**Beam 1**	
6	56 (68·3)
10	25 (30·5)
Missing	1 (1·2)
**Beam 2**	
6	56 (68·3)
10	25 (30·5)
Missing	1 (1·2)
**Beam 3**	
6	5 (6·1)
10	1 (1·2)
Missing	76 (92·7)

**Beam 4**	
6	5 (6·1)
10	1 (1·2)
Missing	76 (92·7)
**Contrast applied**	
Yes	17 (20·7)
No	65 (79·3)
**Dose calculation method used**	
Tables	43 (52·4)
TPS	37 (45·1)
1st calculation check done in RADCALC (Computer)	1 (50·0)
OMP Planning System	1 (50·0)
**Inhomogeneity correction used**	
Yes	18 (22·0)
No	64 (78·0)

*n (%) unless otherwise indicated

Table S4: Baseline characteristics of participants missing vs not missing primary endpoint data

	Complete data up to week 12*	Missing data up to week 12*
	N=149	N=50
**Randomisation time point**		
Before stent	59 (39·6)	16 (32·0)
After stent	90 (60·4)	34 (68·0)
**Age - median (IQR), n**	72·2 (64·8-80·2), 149	75·0 (68·4-83·3), 50
**WHO performance status**		
0	17 (11·4)	3 (6·0)
1	93 (62·4)	27 (54·0)
2	37 (24·8)	17 (34·0)
3	2 (1·3)	3 (6·0)
**Tumour type**		
Adenocarcinoma	96 (64·4)	33 (66·0)
Squamous	51 (34·2)	16 (32·0)
Undifferentiated/other	2 (1·3)	1 (2·0)
**Overall length of primary tumour (endoscopic assessment)**		
Measured length (cm) - median (IQR), n	5·0 (4·0-7·0), 93	5·3 (4·3-7·0), 28
Estimated length (cm) - median (IQR), n	6·0 (5·0-8·0), 42	7·0 (5·5-9·0), 19
Measured/estimated length (cm) - median (IQR), n	6·0 (4·0-8·0), 135	6·0 (5·0-8·0), 47
Missing	14 (9·4)	3 (6·0)
**Alternative method for assessing length**		
PET	9 (6·0)	3 (6·0)
CT	33 (22·1)	13 (26·0)
Barium	0 (0·0)	0 (0·0)
Other	1 (0·7)	0 (0·0)
None	1 (0·7)	0 (0·0)
**Site of predominant tumour**		
Upper	5 (3·4)	1 (2·0)
Middle	34 (22·8)	15 (30·0)
Lower	109 (73·2)	34 (68·0)
If lower, involvement of oesophago-gastric junction (GOJ)	60 (40·3)	16 (32·0)
Unknown	1 (0·7)	0 (0·0)
**Extension across GOJ (if involvement of GOJ)**		
Siewart type 1	32 (21·5)	9 (18·0)
Siewart type 2	26 (17·4)	2 (4·0)
Missing	2 (1·3)	5 (10·0)
**T stage**		
0	1 (0·7)	0 (0·0)
1	1 (0·7)	1 (2·0)
2	7 (4·7)	4 (8·0)
3	87 (58·4)	28 (56·0)
4	45 (30·2)	15 (30·0)
Unknown	2 (1·3)	2 (4·0)
X	6 (4·0)	0 (0·0)
**N stage**		
0	20 (13·4)	7 (14·0)
1	68 (45·6)	24 (48·0)
2	29 (19·5)	11 (22·0)
3	27 (18·1)	5 (10·0)
Unknown	1 (0·7)	2 (4·0)
X	4 (2·7)	1 (2·0)

	Complete data up to week 12*	Missing data up to week 12*
	N=149	N=50
**M stage**		
0	63 (42·3)	24 (48·0)
1	77 (51·7)	22 (44·0)
Unknown	1 (0·7)	2 (4·0)
X	8 (5·4)	2 (4·0)
**Overall stage**		
1-3	70 (47·0)	27 (54·0)
4	79 (53·0)	23 (46·0)

*n (%) unless otherwise indicated

Table S5: Analysis of status and primary endpoint at 12 weeks post stent insertion in the PP population by trial arm

	UC*	EBRT*	Adjusted OR** (95%CI; p-value, n)
	N=90	N=82	
**Incomplete case data at week 12**			
Completely withdrew with no event	2 (2·2)	3 (3·7)	
Died with incomplete data and no event	8 (8·9)	5 (6·1)	
Alive at week 12 with incomplete data and no event	14 (15·6)	10 (12·2)	
**Reasons for complete withdrawal**			
Participant choice	1 (1·1)	2 (2·4)	
Informed by CNS on family's behalf	1 (1·1)	0 (0·0)	
Loss to follow up	0 (0·0)	1 (1·2)	
**Complete case data at week 12**			
Total with complete data	66 (73·3)	64 (78·0)	
Died with complete data	12 (13·3)	14 (17·1)	
Alive at week 12 with complete data	54 (60·0)	50 (61·0)	
**Complete case analysis (death as an event)**			
Number of primary events or deaths	28 (42·4)	26 (40·6)	0·99 (0·45-2·14; 0·972; 130)
**Complete case analysis (death as non-event)**			
Number of primary events	19 (28·8)	19 (29·7)	1·20 (0·53-2·71; 0·666; 130)
**Best case**			
Total with complete data	82 (91·1)	76 (92·7)	
Number of primary events or deaths	32 (39·0)	28 (36·8)	0·96 (0·48-1·93; 0·910; 158)
**Worst case**			
Total with complete data	82 (91·1)	76 (92·7)	
Number of primary events or deaths	45 (54·9)	37 (48·7)	0·80 (0·41-1·58; 0·527; 158)

* n(%) unless otherwise indicated

** Adjusted for randomisation stratification factors

Table S6: Cause of death by trial arm

	UC*	EBRT*
	N=102	N=97
Total deaths	86 (84·3)	82 (84·5)
Oesophageal cancer	79 (91·9)	72 (87·8)
Stent related	2 (2·3)	2 (2·4)
Mediastinitis from suspected tumour perforation	0 (0·0)	1 (1·2)
Acute kidney injury, Bladder cancer	0 (0·0)	1 (1·2)
Cardiovascular	1 (1·2)	1 (1·2)
Chest sepsis	2 (2·3)	1 (1·2)
GI bleed	0 (0·0)	1 (1·2)
Neutropenic Sepsis	0 (0·0)	1 (1·2)
Pulmonary embolism	0 (0·0)	2 (2·4)
Unknown	2 (2·3)	0 (0·0)

*n (%) unless otherwise indicated.

Of the 3 deaths reported through the SAE system, the “fall” was eventually categorised as hospital-acquired pneumonia (after being admitted due to a fall) so is included here under ‘chest sepsis’, the “myocardial infarction” is included here under ‘cardiovascular’, and the “multifocal ischaemic stroke” is included under ‘oesophageal cancer’ as death was felt to be due to progressive oesophageal cancer with ischemic stroke as contributory factor.

Table S7: EORTC QLQ-C30 questionnaire return and missing reason by trial arm

	UC* N=102					EBRT* N=97				
Weeks post stent insertion	Withdrawn/died before form expected (n)	Expected (n)	Actually received (n;%)	Help needed to complete questionnaire (n; %)	Carer completed questionnaire (n;%)	Withdrawn/died before form expected (n)	Expected (n)	Actually received (n;%)	Help needed to complete questionnaire (n; %)	Carer completed questionnaire (n;%)
1	0	102	101 (99·0)	46 (45·5)	5 (5·0)	0	97	96 (99·0)	38 (39·6)	7 (7·3)
4	11	91	77 (84·6)	36 (46·8)	3 (3·9)	6	91	73 (80·2)	28 (38·4)	3 (4·1)
8	23	79	60 (75·9)	32 (53·3)	2 (3·3)	17	80	57 (71·3)	27 (47·4)	4 (7·0)
12	37	65	42 (64·6)	18 (42·9)	0 (0·0)	34	63	47 (74·6)	21 (44·7)	3 (6·4)
16	49	53	36 (67·9)	24 (66·7)	1 (2·8)	45	52	37 (71·2)	20 (54·1)	3 (8·1)
20	57	45	28 (62·2)	19 (67·9)	0 (0·0)	55	42	31 (73·8)	15 (48·4)	2 (6·5)
24	58	44	24 (54·5)	14 (58·3)	1 (4·2)	58	39	25 (64·1)	11 (44·0)	1 (4·0)
28	65	37	20 (54·1)	11 (55·0)	1 (5·0)	70	27	16 (59·3)	10 (62·5)	1 (6·3)
32	72	30	11 (36·7)	9 (81·8)	0 (0·0)	76	21	10 (47·6)	6 (60·0)	2 (20·0)
36	76	26	11 (42·3)	7 (63·6)	0 (0·0)	81	16	9 (56·3)	7 (77·8)	1 (11·1)
40	79	23	12 (52·2)	5 (41·7)	0 (0·0)	82	15	8 (53·3)	5 (62·5)	1 (12·5)
44	85	17	6 (35·3)	3 (50·0)	0 (0·0)	83	14	6 (42·9)	4 (66·7)	1 (16·7)
48	84	18	6 (33·3)	4 (66·7)	0 (0·0)	84	13	4 (30·8)	3 (75·0)	0 (0·0)
52	86	16	3 (18·8)	2 (66·7)	0 (0·0)	85	12	5 (41·7)	2 (40·0)	0 (0·0)

*n (%) unless otherwise indicated

Table S8: EORTC QLQ-OG25 questionnaire return and missing reason by trial arm

	UC*					EBRT*				
	N=102					N=97				
Weeks post stent insertion	Withdrawn/died before form expected (n)	Expected (n)	Actually received (n;%)	Help needed to complete questionnaire (n; %)	Carer completed questionnaire (n;%)	Withdrawn/died before form expected (n)	Expected (n)	Actually received (n;%)	Help needed to complete questionnaire (n; %)	Carer completed questionnaire (n;%)
1	0	102	102 (100·0)	46 (45·1)	5 (4·9)	0	97	97 (100·0)	38 (39·2)	7 (7·2)
4	11	91	78 (85·7)	36 (46·2)	3 (3·8)	6	91	75 (82·4)	28 (37·3)	3 (4·0)
8	23	79	61 (77·2)	32 (52·5)	2 (3·3)	17	80	62 (77·5)	27 (43·5)	4 (6·5)
12	37	65	46 (70·8)	18 (39·1)	0 (0·0)	34	63	49 (77·8)	21 (42·9)	3 (6·1)
16	49	53	41 (77·4)	24 (58·5)	1 (2·4)	45	52	40 (76·9)	20 (50·0)	3 (7·5)
20	57	45	34 (75·6)	19 (55·9)	0 (0·0)	55	42	35 (83·3)	15 (42·9)	2 (5·7)
24	58	44	27 (61·4)	14 (51·9)	1 (3·7)	58	39	26 (66·7)	11 (42·3)	1 (3·8)
28	65	37	21 (56·8)	11 (52·4)	1 (4·8)	70	27	19 (70·4)	10 (52·6)	1 (5·3)
32	72	30	14 (46·7)	9 (64·3)	0 (0·0)	76	21	12 (57·1)	6 (50·0)	2 (16·7)
36	76	26	13 (50·0)	7 (53·8)	0 (0·0)	81	16	10 (62·5)	7 (70·0)	1 (10·0)
40	79	23	12 (52·2)	5 (41·7)	0 (0·0)	82	15	8 (53·3)	5 (62·5)	1 (12·5)
44	85	17	6 (35·3)	3 (50·0)	0 (0·0)	83	14	7 (50·0)	4 (57·1)	1 (14·3)
48	84	18	7 (38·9)	4 (57·1)	0 (0·0)	84	13	5 (38·5)	3 (60·0)	0 (0·0)
52	86	16	4 (25·0)	2 (50·0)	0 (0·0)	85	12	5 (41·7)	2 (40·0)	0 (0·0)

*n (%) unless otherwise indicated

Table S9: Mean or median scores for each quality of life sub-scale or item and WHO performance status over time post stent up to week 16 by trial arm, time and treatment effects and time vs treatment interactions

	Treatment group		Effect				
	UC	EBRT	Time		Treatment		Treatment x time effect (p-value)
	Mean (95%CI), n or Median (IQR), n	Mean (95%CI), n or Median (IQR), n	Adjusted mean difference (95% CI)[1]	p-value	Adjusted mean difference (95% CI)[1]	p-value	
**EORTC-QLQC30/QLQ OG25 domain**							
**Global health (mean)**							
Week 1	49·4 (44·2-54·4), 99	40·2 (35·1-45·2), 95	0·12 (-0·25-0·49)	0·516	-1·99 (-6·59-2·62)	0·398	0·66
Week 4	49·4 (44·5-54·4), 77	43·0 (36·5-49·5), 73					
Week 8	51·8 (45·3-58·3), 57	47·1 (40·4-53·7), 56					
Week 12	54·6 (46·8-62·4), 41	51·4 (45·0-57·9), 46					
Week 16	53·7 (46·7-60·7), 34	49·0 (41·5-56·5), 37					
**Odynophagia (median)**							
Week 1	16·7 (0-50), 101	33·3 (16·7-66·7), 95	-0·65 (-1·06- -·23)	0·002	2·69 (-2·52-7·91)	0·311	0·694
Week 4	16·7 (0-33·3), 77	16·7 (0-50), 74					
Week 8	16·7 (0-33·3), 58	16·7 (0-33·3), 59					
Week 12	16·7 (0-33·3), 43	16·7 (0-50), 46					
Week 16	16·7 (0-33·3), 34	16·7 (0-33·3), 35					
**Dysphagia (median)**							
Week 1	33·3 (16·6-44·4), 102	33·3 (22·2-66·7), 97	-0·75(-1·12- -0·38)	<0·001	4·21 (-0·56-8·98)	0·084	0·013
Week 4	11·1 (0-33·3), 78	22·2 (11·1-44·4), 75					
Week 8	11·1 (0-33·3), 60	11·1 (11·1-33·3), 62					
Week 12	11·1 (0-33·3), 46	11·1 (0-33·3), 49					
Week 16	11·1 (11·1-33·3), 37	11·1 (0-33·3), 37					
**Pain/discomfort OG25 (median)**							
Week 1	16·7 (0-33·3), 102	16·7 (0-50), 94	-0·15 (-0·58-0·29)	0·514	2·08 (-3·50-7·66)	0·466	0·579
Week 4	16·7 (0-33·3), 77	25 (0-33·3), 74					
Week 8	16·7 (0-33·3), 59	33·3 (0-50), 59					
Week 12	16·7 (0-33·3), 44	33·3 (0-66·7), 46					
Week 16	16·7 (0-33·3), 34	33·3 (0-33·3), 35					
**Eating restriction (mean)**							
Week 1	52·9 (46·9-58·9), 102	56·3 (50·6-61·9), 95	-0·45 (-0·87- -0·02)	0·04	1·86 (-3·73-7·47)	0·513	0·555
Week 4	44 (37·2-50·8), 75	47·8 (40·4-55·2), 69					
Week 8	38·6 (31·6-45·6), 59	48·2 (40·3-56·1), 57					
Week 12	38·8 (30·4-47·1), 40	42·6 (33·3-51·8), 46					
Week 16	40·8 (31·4-50·2), 40	42·5 (33·3-51·6), 42					
**Eating in front of others (median)**							
Week 1	0 (0-33·3), 98	0 (0-66·7), 95	-0·37 (-0·86-0·12)	0·138	1·51 (-4·47-7·49)	0·622	0·117

Week 4	0 (0-33·3), 74	0 (0-33·3), 73					
Week 8	0 (0-33·3), 58	0 (0-33·3), 58					
Week 12	0 (0-33·3), 42	33·3 (0-66·7), 45					
Week 16	0 (0-0), 33	33·3 (0-66·7), 35					
**Physical functioning (median)**							
Week 1	26·7 (8·3-53·3), 102	33·3 (13·3-53·3), 95	0·68 (0·34-1·03)	<0·001	1·22 (-3·40-5·84)	0·604	0·643
Week 4	26·7 (13·3-53·3), 77	40 (13·3-60), 73					
Week 8	33·3 (20-46·7), 59	40 (13·3-60), 57					
Week 12	33·3 (13·3-46·7), 41	33·3 (20-53·3), 46					
Week 16	33·3 (13·3-53·3), 30	33·3 (20-58·3), 34					
**Role functioning (mean)**							
Week 1	44·6 (37·4-51·8), 102	48·6 (41·5-55·7), 95	0·41 (-0·09-0·91)	0·11	2·49 (-4·09-9·06)	0·459	0·75
Week 4	45·5 (37·5-53·6), 75	50 (42·0-58·0), 68					
Week 8	43·7 (34·5-52·8), 58	51·9 (42·7-61·0), 54					
Week 12	38·6 (27·6-49·6), 38	47·1 (37·1-57·1), 46					
Week 16	44·9 (33·6-56·3), 36	47·1 (36·1-58·2), 41					
**Emotional functioning (median)**							
Week 1	25 (8·3-41·7), 99	25 (0-50), 95	-0·26 (-0·59-0·06)	0·114	0·50 (-3·65-4·64)	0·815	0·225
Week 4	25 (8·3-41·7), 77	25 (8·3-58·3), 73					
Week 8	25 (0-33·3), 58	20·8 (8·3-41·7), 56					
Week 12	16·7 (8·3-33·3), 41	25 (8·3-33·3), 46					
Week 16	16·7 (0-33·3), 30	20·8 (8·3-50), 34					
**Cognitive functioning (median)**							
Week 1	16·7 (0-33·3), 99	16·7 (0-50), 95	-0·14 (-0·47-0·18)	0·392	-0·35 (-4·64-3·94)	0·874	0·007
Week 4	16·7 (0-33·3), 77	16·7 (0-50), 73					
Week 8	16·7 (0-33·3), 58	16·7 (0-41·7), 56					
Week 12	0 (0-16·7), 41	16·7 (0-33·3), 46					
Week 16	0 (0-16·7), 30	25 (0-50), 34					
**Social functioning (median)**							
Week 1	33·3 (0-66·7), 99	33·3 (0-66·7), 95	0·62 (0·15-1·09)	0·01	2·43 (-3·84-8·71)	0·447	0·351
Week 4	33·3 (0-66·7), 77	33·3 (0-66·7), 73					
Week 8	33·3 (0-66·7), 57	33·3 (8·3-66·7), 56					
Week 12	33·3 (0-50), 41	33·3 (16·7-66·7), 46					
Week 16	33·3 (0-66·7), 30	33·3 (16·7-66·7), 34					
**Fatigue (median)**							
Week 1	44·4 (22·2-77·8), 102	55·6 (33·3-77·8), 95	0·37 (-0·02-0·77)	0·065	3·50 (-1·68-8·68)	0·186	0·522
Week 4	44·4 (33·3-66·7), 77	66·7 (33·3-88·9), 73					
Week 8	44·4 (33·3-66·7), 59	44·4 (33·3-77·8), 57					
Week 12	44·4 (22·2-66·7), 41	55·6 (33·3-66·7), 46					

Week 16	44·4 (33·3-66·7), 30	44·4 (33·3-77·8), 34					
**Nausea/vomiting (median)**							
Week 1	16·7 (0-50), 102	33·3 (16·7-66·7), 95	-0·54 (-0·98- -0·10)	0·017	5·60 (0·11-11·08)	0·046	0·195
Week 4	16·7 (0-33·3), 77	33·3 (0-50), 73					
Week 8	16·7 (0-33·3), 59	16·7 (0-33·3), 57					
Week 12	16·7 (0-33·3), 41	16·7 (0-33·3), 46					
Week 16	0 (0-33·3), 30	16·7 (0-33·3), 34					
**Pain C30 (median)**							
Week 1	33·3 (16·7-66·7), 102	50 (16·7-66·7), 95	-0·56 (-1·01- -0·11)	0·015	2·39 (-3·25-8·03)	0·406	0·005
Week 4	33·3 (16·7-50), 77	33·3 (16·7-66·7), 73					
Week 8	16·7 (0-33·3), 58	33·3 (16·7-66·7), 57					
Week 12	16·7 (0-50), 41	33·3 (16·7-66·7), 46					
Week 16	16·7 (0-50), 30	50 (16·7-83·3), 34					
**Dyspnoea (median)**							
Week 1	33·3 (0-33·3), 102	33·3 (0-66·7), 94	0·17 (-0·25-0·59)	0·427	2·50 (-3·03-8·02)	0·376	0·169
Week 4	33·3 (0-33·3), 77	33·3 (0-66·7), 73					
Week 8	33·3 (0-33·3), 59	33·3 (0-33·3), 57					
Week 12	33·3 (0-33·3), 41	33·3 (0-66·7), 46					
Week 16	33·3 (0-33·3), 30	33·3 (0-66·7), 34					
**Insomnia (mean)**							
Week 1	37·6 (30·8-44·4), 102	44·2 (36·7-51·7), 95	-0·70 (-1·21- -0·18)	0·009	6·76 (-0·10-13·42)	0·047	0·688
Week 4	32·9 (25·2-40·6), 74	42·3 (32·7-51·8),67					
Week 8	25·9 (17·6-34·1), 58	36·4 (26·4-46·5), 54					
Week 12	21·6 (11·4-31·8), 37	31·9 (21·8-41·9), 45					
Week 16	29·6 (18·9-40·3), 36	37·5 (25·6-49·4), 40					
**Appetite loss (mean)**							
Week 1	50·8 (43·6-58·0), 101	62·8 (55·4-70·2), 94	-0·30 (-0·90-0·30)	0·56	6·42 (-1·18-14·02)	0·098	0·612
Week 4	52·9 (45·1-60·7), 75	55·9 (46·6-65·2), 68					
Week 8	37·9 (29·0-46·8), 58	55·8 (46·4-65·1), 55					
Week 12	45·9 (33·0-58·9), 37	47·8 (36·9-58·8), 46					
Week 16	41·7 (31·1-52·2), 36	49·6 (38·8-60·4), 41					
**Constipation (mean)**							
Week 1	42·4 (35·9-48·9), 99	41·1 (34·2-47·9), 95	-0·95 (-1·49- -0·41)	0·003	-4·33 (-10·78-2·12)	0·24	0·009
Week 4	48·0 (40·3-55·7), 75	44·1 (35·1-53·1), 68					
Week 8	30·4 (22·5-38·3), 57	35·8 (27·2-44·4), 54					
Week 12	22·8 (13·6-32·0), 38	37·7 (28·7-46·7), 46					
Week 16	27·8 (18·6-36·9), 36	41·5 (31·0-51·9), 41					
**Diarrhoea (median)**							
Week 1	0 (0-0), 98	0 (0-33·3), 95	0·15 (-0·28-0·59)	0·493	2·81 (-2·27-7·89)	0·279	0·147

Week 4	0 (0-0), 77	0 (0-33·3), 72					
Week 8	0 (0-33·3), 57	0 (0-0), 56					
Week 12	0 (0-33·3), 41	0 (0-0), 45					
Week 16	0 (0-0), 30	0 (0-33·3), 34					
**Financial difficulties (median)**							
Week 1	0 (0-0), 99	0 (0-33·3), 94	-0·15 (-0·47-0·17)	0·4	-0·27 (-4·46-3·91)	0·899	0·445
Week 4	0 (0-0), 76	0 (0-0), 73					
Week 8	0 (0-0), 56	0 (0-0), 56					
Week 12	0 (0-0), 41	0 (0-0), 46					
Week 16	0 (0-0), 29	0 (0-33·3), 34					
**Body image (median)**							
Week 1	0 (0-33·3), 101	0 (0-66·7), 94	-0·15 (-0·66-0·36)	0·92	-2·23 (-8·66-4·20)	0·496	0·011
Week 4	0 (0-33·3), 75	0 (0-33·3), 74					
Week 8	0 (0-33·3), 59	33·3 (0-66·7), 59					
Week 12	0 (0-33·3), 44	0 (0-66·7), 45					
Week 16	0 (0-0), 33	33·3 (0-66·7), 34					
**Reflux (median)**							
Week 1	33·3 (0-50), 102	33·3 (0-66·7), 95	-0·55 (-0·96- -0·15)	0·007	0·72 (-4·47-5·92)	0·785	0·825
Week 4	16·7 (0-33·3), 77	33·3 (0-50), 74					
Week 8	16·7 (0-50), 59	16·7 (0-33·3), 59					
Week 12	16·7 (0-50), 44	16·7 (0-50), 46					
Week 16	8·33 (0-33·3), 34	33·3 (0-50), 35					
**Anxiety (mean)**							
Week 1	55·4 (49·2-61·6), 102	51·2 (44·6-57·9), 95	-0·91 (-1·32- -0·50)	<0·001	-2·87 (-8·45-2·71)	0·313	0·002
Week 4	49·6 (42·4-56·7), 75	47·6 (39·3-55·9), 69					
Week 8	45·5 (38·1-52·8), 59	44·2 (36·1-52·2), 57					
Week 12	38·8 (29·4-48·1), 40	49·3 (40·1-58·4), 46					
Week 16	40 (30·5-49·5), 40	46·8 (35·9-57·8), 42					
**Dry mouth (median)**							
Week 1	33·3 (0-66·7), 100	33·3 (0-66·7), 95	-0·58 (-1·08- 0·09)	0·022	0·64 (-5·61-6·89)	0·84	0·816
Week 4	33·3 (0-66·7), 77	33·3 (0-66·7), 74					
Week 8	0 (0-33·3), 59	33·3 (0-66·7), 59					
Week 12	16·7 (0-33·3), 44	33·3 (0-33·3), 43					
Week 16	0 (0-33·3), 34	33·3 (0-66·7), 34					
**Trouble with taste (median)**							
Week 1	0 (0-33·3), 101	33·3 (0-66·7), 95	0·44 (-0·05-0·94)	0·079	1·08 (-5·39-7·55)	0·744	0·547
Week 4	0 (0-66·7), 76	0 (0-66·7), 73					
Week 8	0 (0-33·3), 59	0 (0-66·7), 59					
Week 12	16·7 (0-50), 44	33·3 (0-33·3), 46					

Week 16	0 (0-33·3), 33	33·3 (0-66·7), 34					
**Trouble swallowing saliva (median)**							
Week 1	0 (0-33·3), 102	0 (0-33·3), 95	-0·79 (-1·22- -0·36)	<0·001	-1·84 (-7·31-3·63)	0·511	0·025
Week 4	0 (0-33·3), 76	0 (0-33·3), 74					
Week 8	0 (0-33·3), 59	0 (0-33·3), 59					
Week 12	0 (0-33·3), 44	0 (0-33·3), 46					
Week 16	0 (0-0), 34	0 (0-33·3), 35					
**Choked when swallowing (median)**							
Week 1	0 (0-33·3), 102	0 (0-33·3), 95	-0·49 (-0·86-0·11)	0·011	-0·74 (-5·29-3·80)	0·749	0·321
Week 4	0 (0-0), 77	0 (0-33·3), 74					
Week 8	0 (0-0), 59	0 (0-33·3), 59					
Week 12	0 (0-0), 44	0 (0-0), 46					
Week 16	0 (0-0), 34	0 (0-0), 35					
**Trouble with coughing (median)**							
Week 1	33·3 (0-33·3), 101	33·3 (0-33·3), 95	-0·28 (-0·71-0·15)	0·202	-0·62 (-5·94-4·70)	0·82	0·37
Week 4	33·3 (0-33·3), 77	33·3 (0-33·3), 74					
Week 8	33·3 (0-33·3), 59	33·3 (0-33·3), 57					
Week 12	33·3 (0-33·3), 43	33·3 (0-33·3), 46					
Week 16	33·3 (0-33·3), 34	33·3 (0-33·3), 34					
**Trouble talking (median)**							
Week 1	0 (0-0), 99	0 (0-33·3), 94	0·00 (-0·33-0·33)	0·99	0·56 (-3·55-4·67)	0·79	0·487
Week 4	0 (0-0), 75	0 (0-33·3), 74					
Week 8	0 (0-0), 59	0 (0-0), 58					
Week 12	0 (0-0), 44	0 (0-0), 45					
Week 16	0 (0-0), 34	0 (0-33·3), 35					
**Weight loss (mean)**							
Week 1	40·6 (33·2-48·0), 101	41·8 (34·0-49·5), 95	-0·56 (-1·06-0·05)	0·03	-1·01 (-7·71-5·69)	0·767	0·053
Week 4	43·7 (35·3-52·1), 74	45·4 (36·6-54·2), 69					
Week 8	32·8 (23·9-41·7), 59	36·3 (27·6-45·0), 56					
Week 12	30·0 (18·7-41·3), 40	42·8 (33·3-52·2), 46					
Week 16	30·8 (19·2-42·5), 40	42·1 (30·8-53·3), 42					
**Hair loss (median)**							
Week 1	0 (0-0), 17	0 (0-33·3), 21	0·10 (-0·58-0·77)	0·781	0·61(-7·98-9·20)	0·889	0·16
Week 4	0 (0-0), 13	0 (0-16·7), 16					
Week 8	0 (0-33·3), 15	0 (0-33·3)11					
Week 12	33·3 (0-33·3), 10	16·7 (0-33·3), 12					
Week 16	0 (0-0), 11	0 (0-33·3), 8					
**WHO performance status (mean)**							
Week 1	1·30 (1·16-1·45), 102	1·32 (1·19-1·45), 97	0·03 (0·02-0·04)	<0·001	0·06 (-0·09-0·20)	0·457	0·565

Week 4	1·47 (1·30-1·64), 76	1·61 (1·44-1·79), 75					
Week 8	1·57 (1·34-1·80), 63	1·73 (1·52-1·95), 60					
Week 12	1·44 (1·21-1·67), 48	1·55 (1·35-1·75), 49					
Week 16	1·52 (1·27-1·77), 42	1·77 (1·55-2·00), 44					

Table S10: Additional palliative radiotherapy given, by trial arm

	UC*	EBRT*
	N=102	N=97
**Additional palliative radiotherapy - N(%)**		
Yes**	20 (19·6)	9 (9·3)
No	82 (80·4)	88 (90·7)
**If yes, Organ/Region - N(%)**		
Oesophagus***	16 (80·0)	2 (22·2)
Bilateral sub clavian fossa	1 (5·0)	0 (0·0)
Brain	1 (5·0)	1 (11·1)
Femur	1 (5·0)	0 (0·0)
Gastro-oesophageal junction	1 (5·0)	0 (0·0)
L1-L3 vertebrae and left pelvis	0 (0·0)	1 (11·1)
L4-S1	0 (0·0)	1 (11·1)
Left lung	1 (5·0)	0 (0·0)
Left supraclavicular fossa	0 (0·0)	1 (11·1)
Lumbar spine	0 (0·0)	1 (11·1)
Posterior ribs	0 (0·0)	1 (11·1)
Right hip	1 (5·0)	0 (0·0)
Scapula	0 (0·0)	2 (22·2)
Total dose (Gy) - median(IQR), n	20·0 (8·0-30·0), 19	8·0 (8·0-20·0), 9
Total fractions - median(IQR), n	5·0 (3·0-10·0), 19	1·0 (1·0-5·0), 9

* n(%) unless otherwise indicated
** post hoc RR 0·47 [95% CI 0·23–0·99] p=0.039
*** post hoc RR 0·28 [95% CI 0·08–0·96] p=0.0030

Table S11: Post-stent insertion chemotherapy

	UC*	EBRT*
	N=102	N=97
**MDT intention to give chemotherapy**		
At baseline, MDT intend to give chemotherapy	36 (35·3)	34 (35·1)
Of those, what proportion did get chemotherapy**	29 (80·6)	15 (44·1)
**Received chemotherapy for oesophageal cancer given post stent insertion**		
Yes	33 (32·4)	17 (16·7)
No	68 (66·7)	79 (77·5)
Missing	1 (1·0)	1 (1·0)
If yes, which?***		
ECF	2 (6·1)	1 (5·9)
CAP	2 (6·1)	0 (0·0)
EOX	13 (39·4)	8 (47·1)
5FU	1 (3·0)	0 (0·0)
Other	31 (93·9)	13 (76·5)
1) ECX x 3cycle. 2) Docetaxel x3 cycles. 3) Irinotecan.	0 (0·0)	1 (5·9)
315 TRAS, C2+PLAT	1 (3·0)	0 (0·0)
CX	4 (12·1)	1 (5·9)
CX + Herceptin	6 (18·2)	2 (11·8)
Capecitabine	2 (6·1)	0 (0·0)
Carboplatin	1 (3·0)	0 (0·0)
Carboplatin + Epirubicin	1 (3·0)	0 (0·0)
Carboplatin + Herceptin	0 (0·0)	2 (11·8)
Carboplatin + Paclitaxel	1 (3·0)	1 (5·9)
Cisplatin	2 (6·1)	1 (5·9)
Docetaxel	4 (12·1)	1 (5·9)
Durvalumab	1 (3·0)	0 (0·0)
ECX	0 (0·0)	1 (5·9)
Epirubicin + Oxaliplatin	1 (3·0)	0 (0·0)
Folfiri	1 (3·0)	0 (0·0)
Herceptin	0 (0·0)	1 (5·9)
Irinotecan	0 (0·0)	1 (5·9)
OxCap	2 (6·1)	0 (0·0)
Oxaliplatin	2 (6·1)	0 (0·0)
Paclitaxel	1 (3·0)	0 (0·0)
Paclitaxel and Ramucirumab	1 (3·0)	0 (0·0)
Raltitrexed Oxaliplatin	0 (0·0)	1 (5·9)
Intended number of cycles-median(IQR), n	6·0 (4·0-6·0), 29	6·0 (4·0-6·0), 16
Intended number of cycles missing	4 (12·1)	1 (5·9)
Number of cycles given-median(IQR), n	3·0 (2·0-4·0), 33	4·0 (3·0-4·0), 16
Number of cycles given missing	0 (0·0)	1 (5·9)

*n (%) unless otherwise indicated
** post hoc RR 0·55 [95% CI 0·36–0·83] p= 0.0016
***Some patients had more than one

Table S12: Results of the cost-utility analysis for 1,000 simulated patients (including 500 patients in each of the two trial arms)

	UC	EBRT	Difference
**Total cost**	£2,313,998	£3,078,427	£764,429
**Average cost per patient**	£4,628	£6,157	£1,529
**Total QALYs**	55·32	53·93	-1·3919
**Average QALYs per patient**	0·1106	0·108	-0·00278
**ICER (Cost per QALY gained)**	**Dominated (-£549,200)**		

Table S13: Results of the one-way sensitivity health economic analyses

SA-ID	Parameter	Change	ICER	
			12 weeks	12 months
Base case	n/a	n/a	Dominated	Dominated
SA1	Costs	-10%	Dominated	Dominated
SA2	Costs	-20%	Dominated	Dominated
SA3	Costs	-30%	Dominated	Dominated
SA4	Costs	+10%	Dominated	Dominated
SA5	Costs	+20%	Dominated	Dominated
SA6	Costs	+30%	Dominated	Dominated
SA7	Costs	Intervention cost micro-costed	Dominated	Dominated
SA8	Utilities	-10%	Dominated	Dominated
SA9	Utilities	-20%	Dominated	Dominated
SA10	Utilities	-30%	Dominated	Dominated
SA11	Utilities	+10%	Dominated	Dominated
SA12	Utilities	+20%	Dominated	Dominated
SA13	Utilities	+30%	Dominated	Dominated
SA14	All parameters	Complete cases used	Dominated	Dominated
SA15	All parameters	All available cases used	Dominated	Dominated
SA16	All parameters post 12 weeks	Weeks 13 to 16 used	Dominated	Dominated
SA17	All parameters post 12 weeks	Weeks 13 to 28 used	Dominated	Dominated

Table S14: Baseline prior chemotherapy

	UC*	EBRT*
	N=102	N=97
**Previous chemotherapy given**		
No	87 (85·3)	74 (76·3)
Yes	15 (14·7)	23 (23·7)
EOX	6 (5·9)	7 (6·9)
ECX	4 (3·9)	3 (2·9)
Cisplatin+Capecitabine	1 (1·0)	3 (2·9)
CX; OxCap	0 (0·0)	2 (2·0)
OxCap	2 (2·0)	0 (0·0)
Carboplatin+Capecitabine+Epirubicin	0 (0·0)	1 (1·0)
Carboplatin+Paclitaxel	0 (0·0)	1 (1·0)
Cisplatin; 5FU	0 (0·0)	1 (1·0)
Cisplatin+Epirubicin	0 (0·0)	1 (1·0)
CX	1 (1·0)	0 (0·0)
CX+Herceptin; Docetaxel	0 (0·0)	1 (1·0)
Docetaxel; Irinotecan	0 (0·0)	1 (1·0)
ECF	0 (0·0)	1 (1·0)
ECX neoadjuvant; EOX	0 (0·0)	1 (1·0)
EOX; Docetaxel	1 (1·0)	0 (0·0)
**If had prior chemotherapy**		
Intended number of cycles - median(IQR), n	6·0 (4·0-8·0), 15	4·0 (3·0-6·0), 21
Intended number of cycles missing	0 (0·0)	2 (8·7)
Number of prior chemotherapy cycles given – median (IQR), n	3·0 (2·0-6·0), 15	4·0 (3·0-6·0), 23

*n (%) unless otherwise indicated

Table S15: List of recruiting sites

Site	Principal Investigator	Patients randomised
Ninewells Hospital, NHS Tayside Dundee	Dr Douglas Adamson	61
University Hospitals Bristol NHS Foundation Trust Royal Infirmary	Professor Jane Blazeby	27
Weston Park, Sheffield Teaching Hospitals NHS Foundation Trust	Dr Jonathan Wadsley	18
University Hospital of Wales, Cardiff and Vale University Health Board	Dr Anthony Byrne	12
University Hospital Coventry, Coventry and Warwickshire NHS Trust	Dr Martin Scott-Brown	10
University Hospital Llandough, Cardiff and Vale University Health Board	Dr Anthony Byrne	9
Musgrove Park Hospital, Taunton and Somerset NHS Foundation Trust	Dr Julie Walther	9
Basildon and Thurrock University Hospitals Foundation NHS Trust	Dr Olivia Chan	8
St Mary's Hospital, Imperial College Healthcare	Dr D A Power	8
Nottingham University Hospitals NHS Trust	Dr Ravi Vohra/Dr Eleanor James	7
Royal Glamorgan Hospital, Cwm Taff Morgannwg University Health Board	Dr Paul Shaw	7
Weston Super Mare Hospital, Weston Area Health Trust	Dr Serena Hilman	7
Worthing Hospital, Western Sussex Hospitals NHS Foundation trust	Dr Angus Robinson/Dr Elizabeth Selvaduri	6
Conquest Hospital Hastings, East Sussex Healthcare NHS Trust	Dr Angus Robinson	5
Royal Sussex Hospital Brighton, East Sussex Healthcare NHS Trust	Dr Angus Robinson	4
University Hospital Southampton Foundation Trust	Dr Andrew Bateman	3
George Eliot Hospital NHS Trust	Dr Martin Scott-Brown	3
Kent and Canterbury Hospital, East Kent Hospitals NHS Foundation Trust	Dr Mathilda Cominos	3
King's Mill Hospital, Sherwood Forest Hospitals NHS Foundation Trust	Dr Eleanor James	3
Doncaster and Bassetlaw Hospitals Hospitals NHS Foundation Trust	Dr Jonathan Wadsley	3
Southend University Hospital Hospitals NHS Foundation Trust	Dr Olivia Chan/Dr David Tsang	3
Royal Gwent Hospital, Aneurin Bevan University Health Board	Professor Ashraf Rasheed	2
James Cook Hospital, South Tees Hospitals NHS Foundation Trust	Dr Nick Wadd	2
		220

Supplementary figures

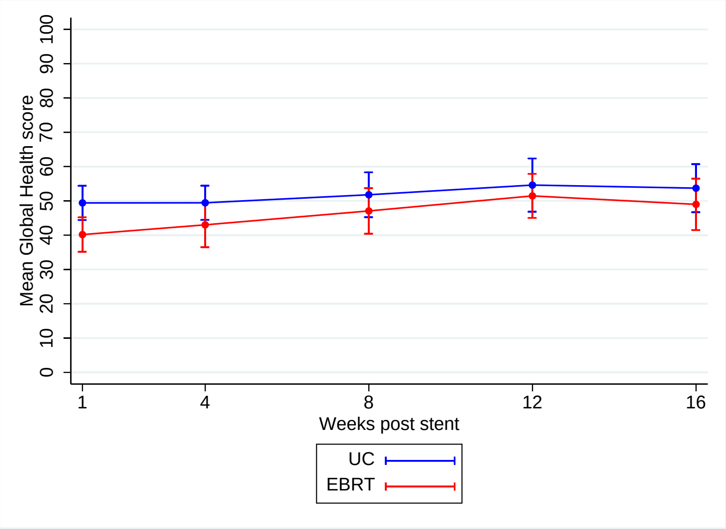

Figure S1: Mean Global Health scores (EORTC-QLQ-C30) and 95% confidence intervals by time and treatment group

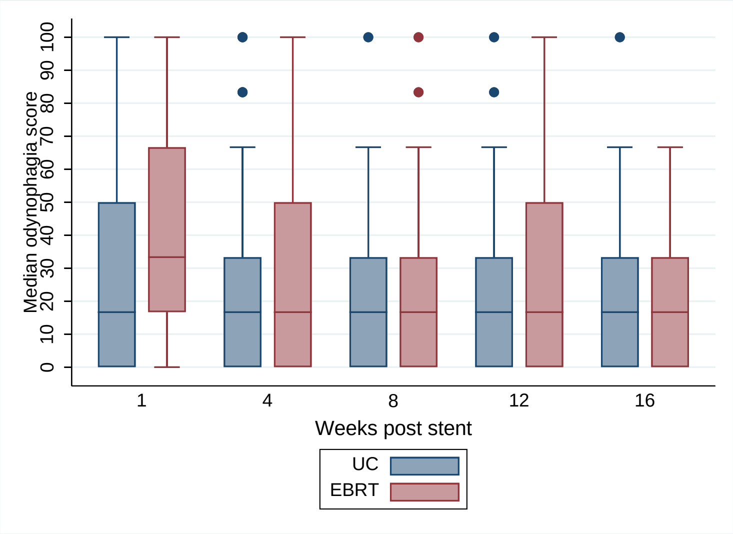

Figure S2: Box plots of odynophagia scores by time and treatment group

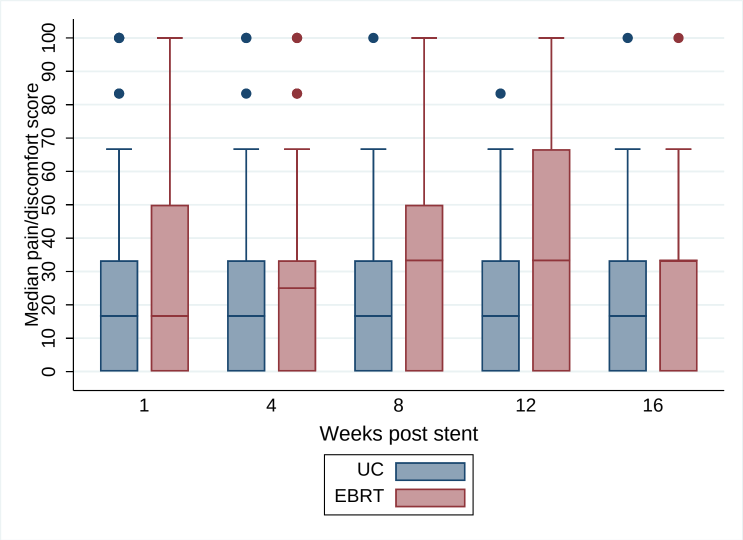

Figure S3: Box plots of pain/discomfort scores (QLQ-OG25 questionnaire) by time and treatment

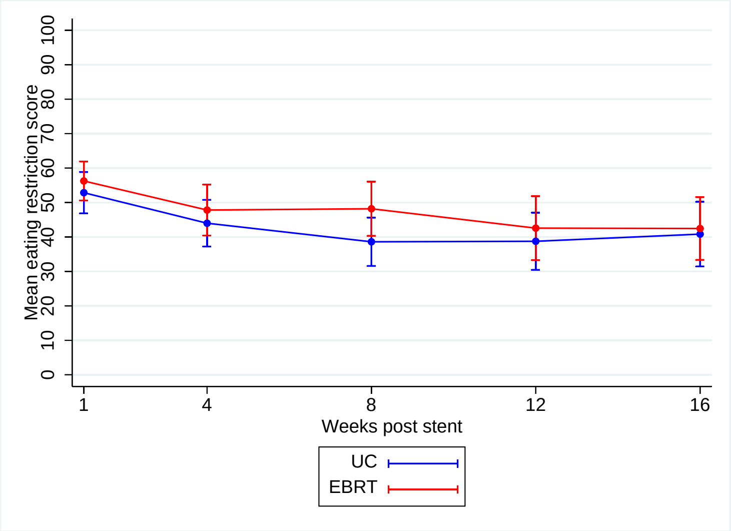

Figure S4: Mean eating restrictions scores and 95% confidence intervals by time and treatment group

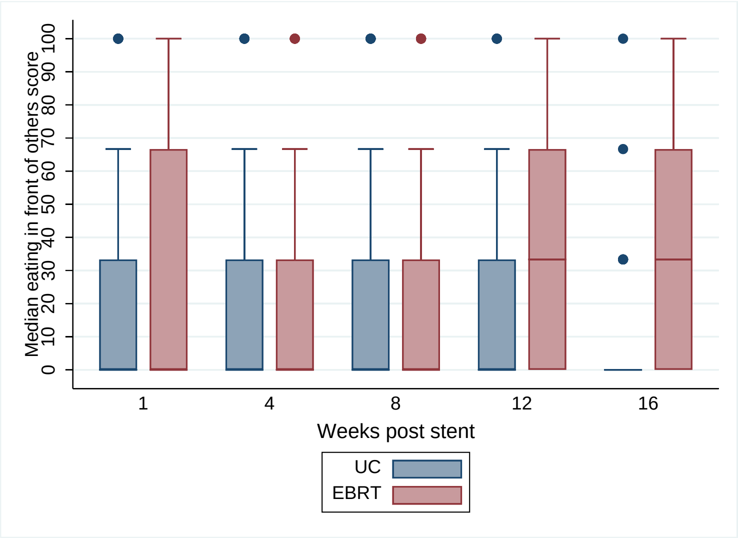

Figure S5: Box plots of “eating in front of others” scores by time and treatment group

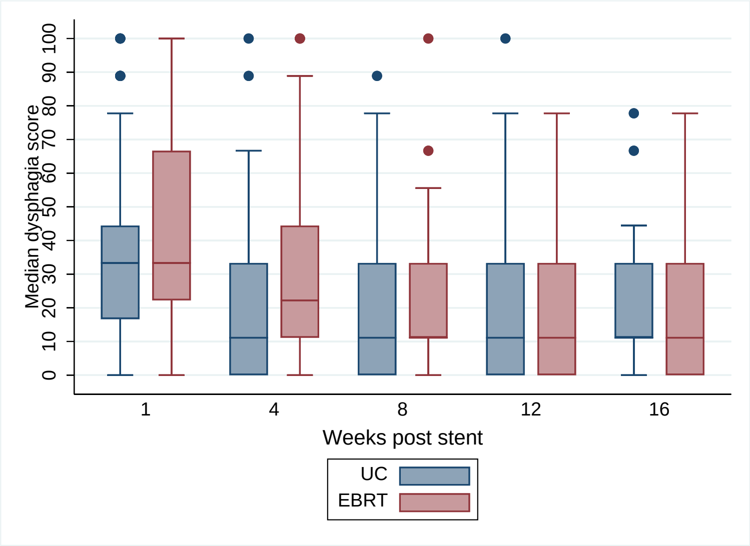

Figure S6: Box plots of dysphagia scores by time and treatment group

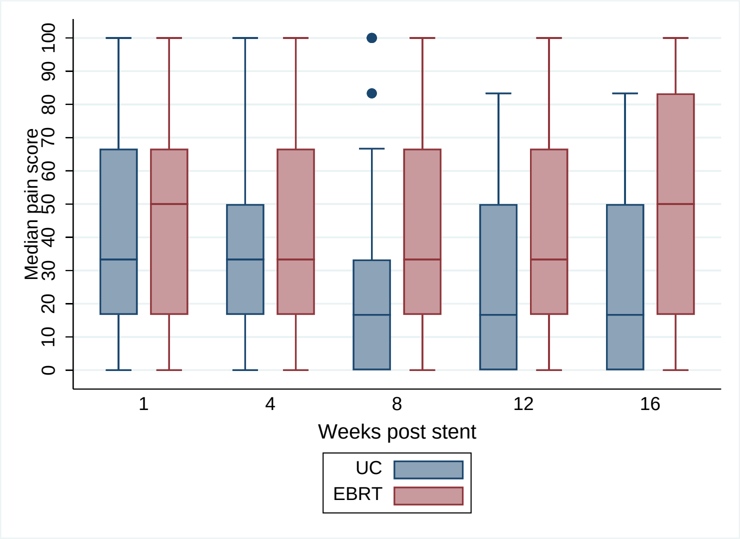

Figure S7: Box plots of pain scores (QLQ-C30) by time and treatment group

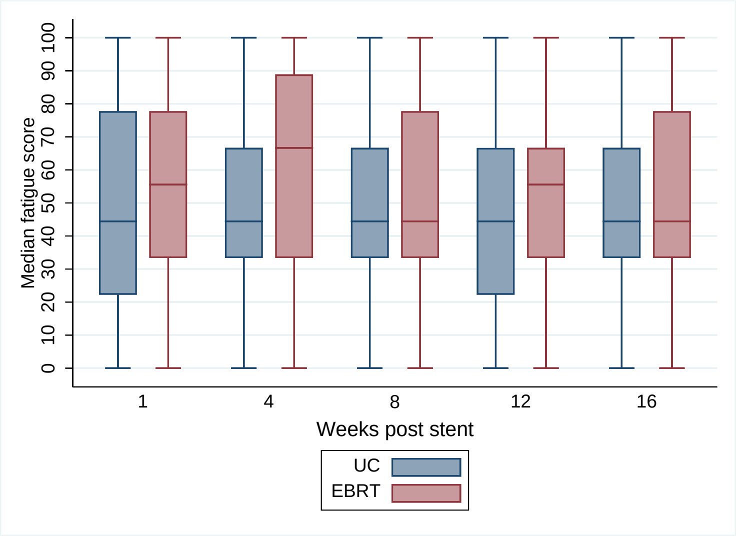

Figure S8: Box plots of fatigue scores by time and treatment group

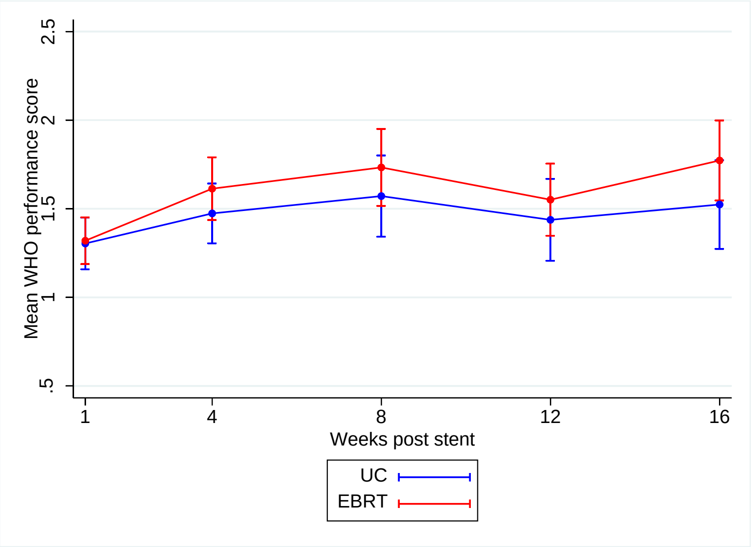

Figure S9: Mean WHO performance status scores and 95% confidence intervals by time and treatment group

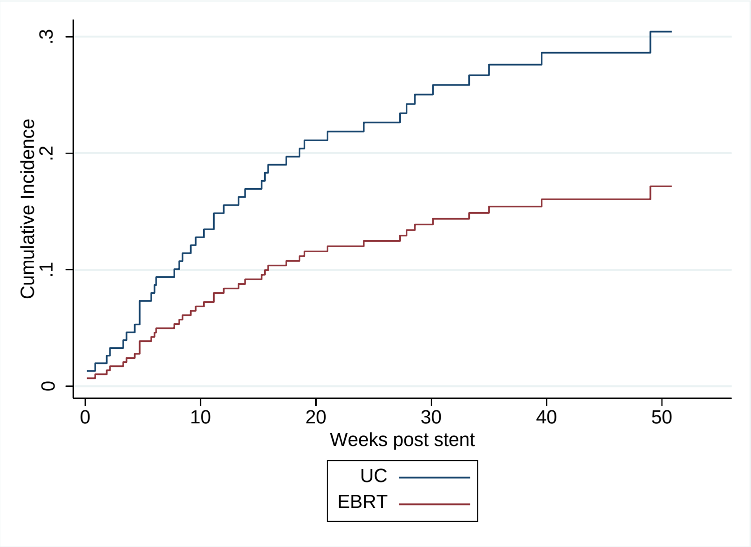

Figure S10: Cumulative incidence plots of time to first upper GI related bleed event (up to week 52)

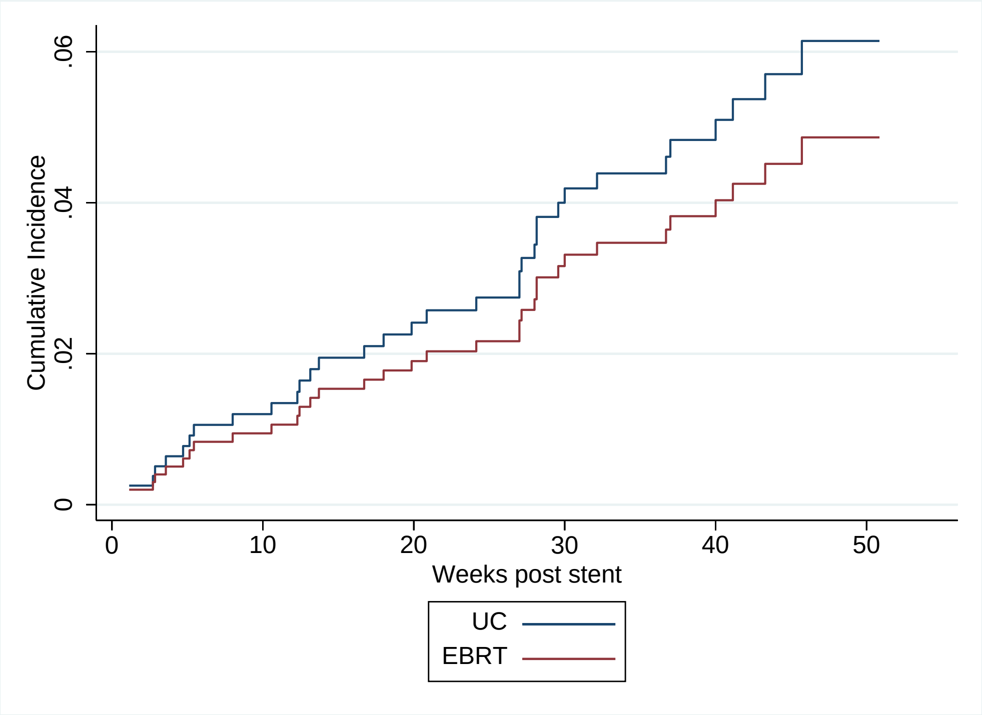

Adjusted SHR 0·79 (95%CI 0·37-1·66; two-sided p-value 0·529; n=199).
Median time to first dysphagia-related stent complication or re-intervention event (weeks):

Stent only arm 45·7 (95%CI 37·0-·; n=102)

Stent plus radiotherapy arm 58·9 (95%CI 36·7-; n=97)

Figure S11: Cumulative Incidence Function plot of first dysphagia-related stent complication or re-intervention by trial arm (up to week 52)

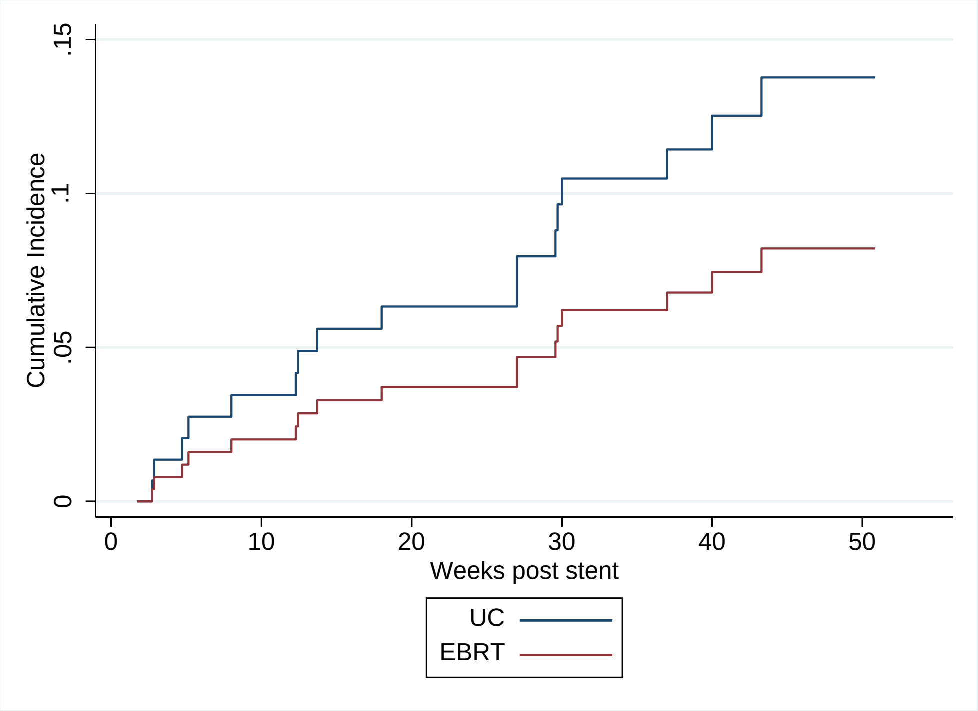

Adjusted SHR 0·58 (95%CI 0·23-1·46; two-sided p-value 0·246; n=199).
Median time to first additional stent insertion (weeks):

Stent only arm was not reached

Stent plus radiotherapy arm 59·7 (95%CI 43·3-not reached)

Figure S12: Cumulative Incidence Function plot of first additional stent insertion by trial arm, with death as a competing risk (up to week 52)

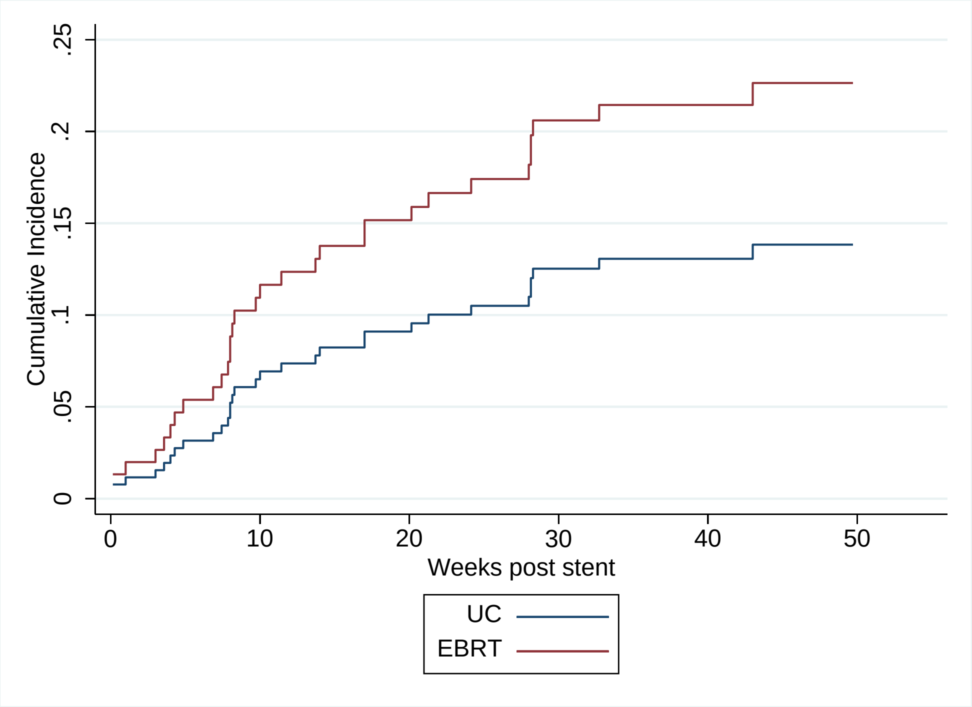

Adjusted SHR 1·72 (95%CI 0·86-3·47; two-sided p-value 0·126; n=199)
Median time to first endoscopy (weeks) was not reached in either arm.

Figure S13: Cumulative Incidence Function plot of first repeat endoscopy by trial arm (up to week 52)

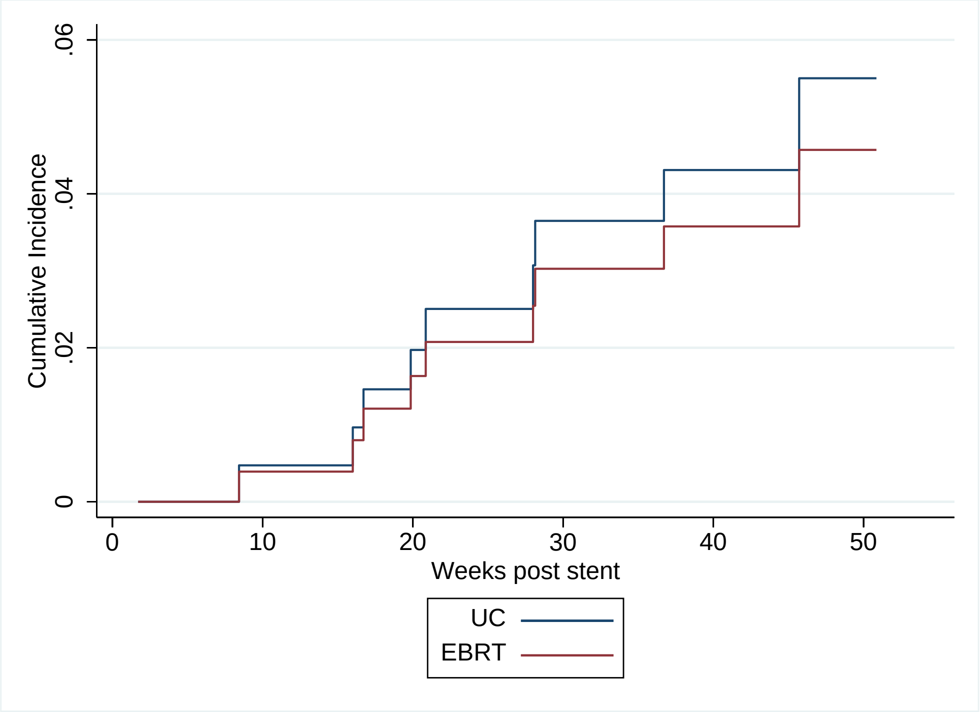

Adjusted SHR 0·83 (95%CI 0·21-3·24; two-sided p-value 0·785; n=199).
Median time to first overgrowth or undergrowth of stent (weeks) was not reached in either arm.

Figure S14: Cumulative Incidence Function plot of first overgrowth or undergrowth of stent by trial arm, with death as a competing risk (up to week 52)

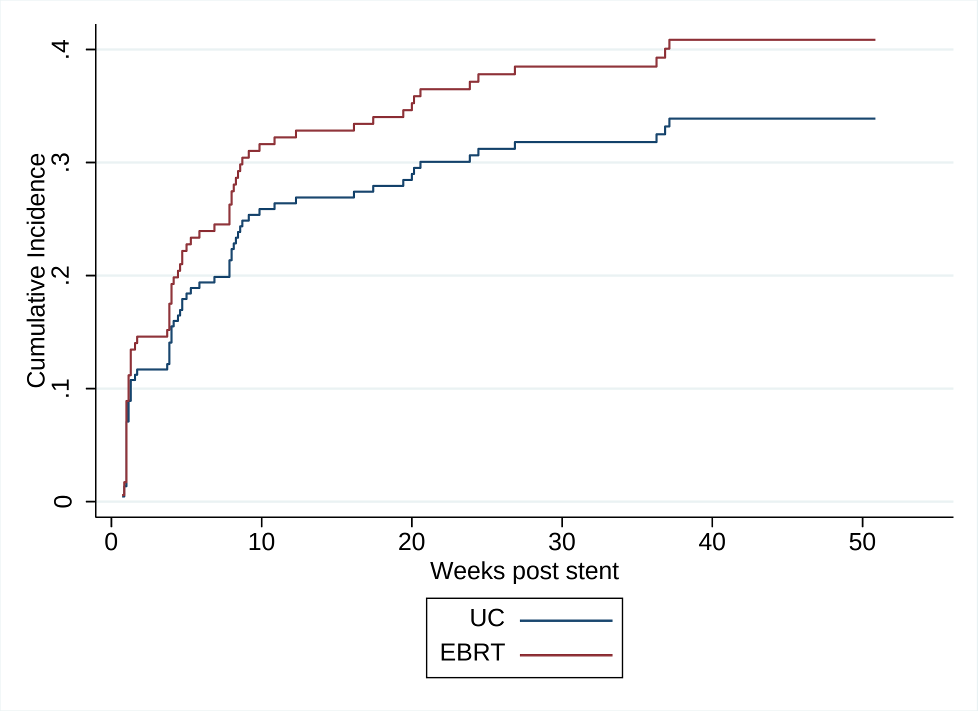

Adjusted SHR 1·27 (95%CI 0·80-2·03; two-sided p-value 0·318; n=199).
Median time to first stent-related pain event (weeks) was not reached vs 36·3 (95%CI 20·0-not reached) stent vs stent plus radiotherapy.

Figure S15: Cumulative Incidence Function plot of first Grade 2+ stent-related pain event by trial arm, with death as a competing risk

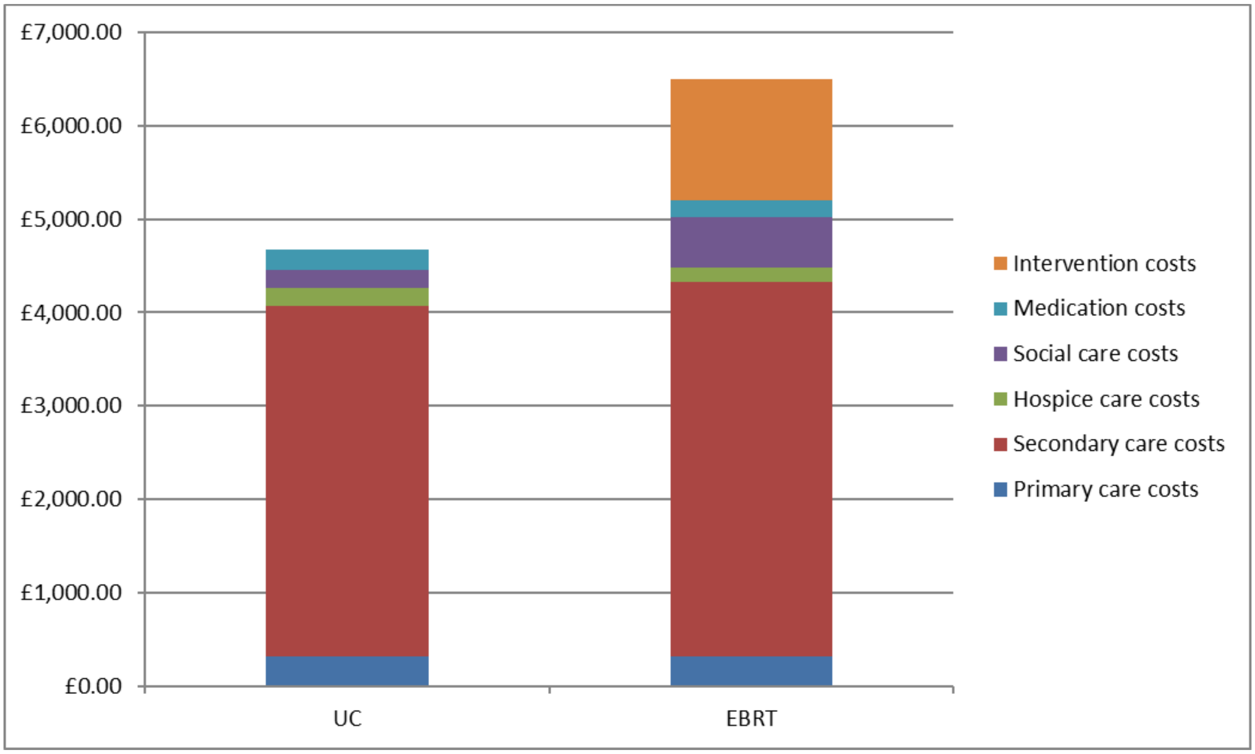

Figure S16: Mean health care costs in the modified ITT population in the 12-week follow up period

A)

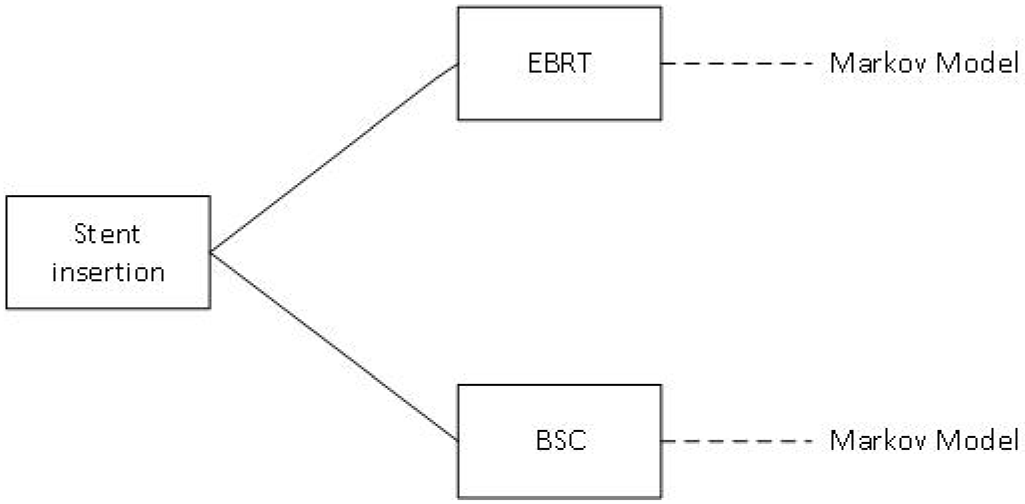

B)

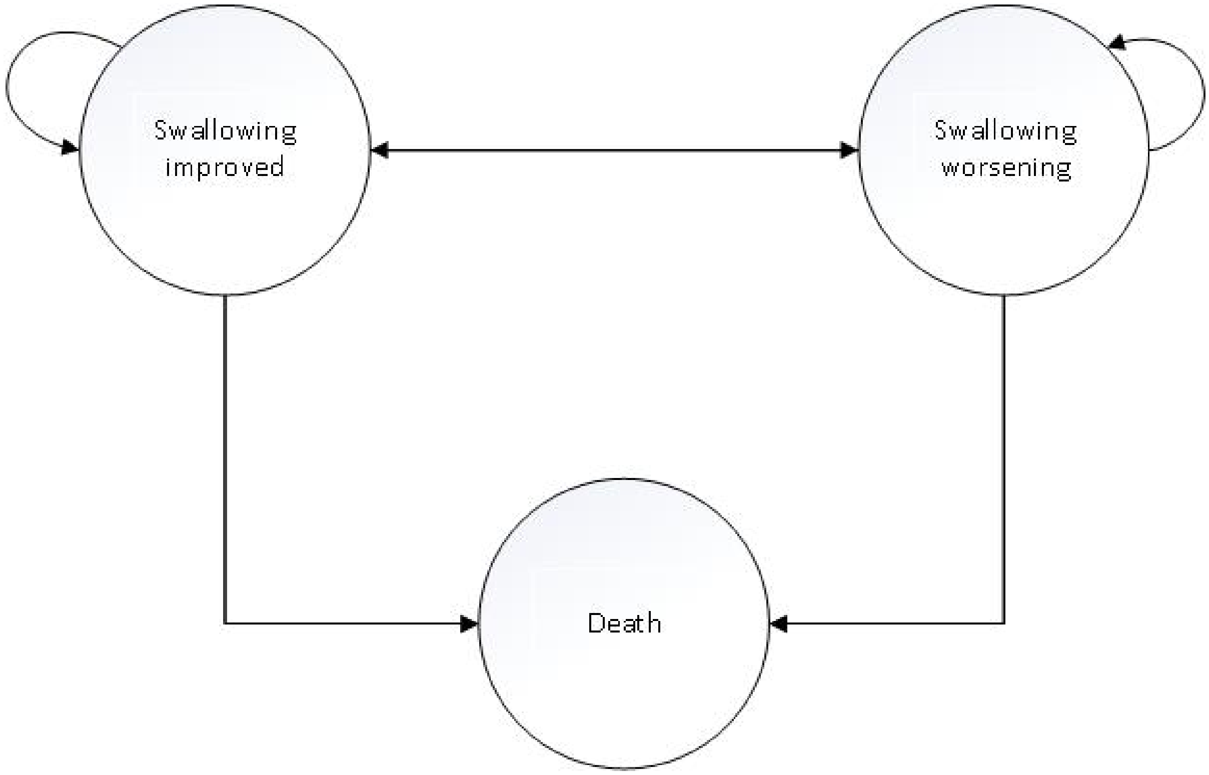

Figure S17: Schematic of the decision-analytic model constructed to calculate the cost-effectiveness of EBRT.

A) Decision tree for weeks 0 to 4; B) Markov model for weeks 5 to 12
